# Reception of conspecific cues alters testicular gene expression and improves fertility in boreal chorus frogs (*Pseudacris maculata*)

**DOI:** 10.1038/s41598-026-43613-6

**Published:** 2026-03-11

**Authors:** Jeffrey P. Ethier, Hyojin Lee, Stacey A. Robinson, Vance L. Trudeau

**Affiliations:** 1 Department of Biology, University of Ottawa, Ottawa, ON Canada; 2 Ecotoxicology and Wildlife Health Division, Wildlife and Landscape Science Directorate, Science and Technology Branch, Environment and Climate Change Canada, Burlington, ON Canada

**Keywords:** Reproduction, Animal behaviour, RNA-sequencing, Social modulation, Gene expression, Developmental biology, Ecology, Ecology, Evolution, Physiology, Zoology

## Abstract

Evidence for the social modulation of reproduction in vertebrates is established but poorly defined for many taxa despite the link between social cues and endocrine function. It is unclear if physiological effects, such as increased sex hormone concentration or gamete maturation, impact reproductive outcomes, such as fertilization rates or viability of offspring. We investigated the effect of conspecific signals on the calling behaviour, reproductive output, and gonadal gene expression in the boreal chorus frogs (*Pseudacris maculata*). Using breeding trials in a captive setting, we found that broadcasts of conspecific calls nearly doubled the duration of calling activity and increased the proportion of viable eggs by over 13%. We then used RNA-sequencing techniques to demonstrate exposure to broadcasts of chorus behaviour increased the expression of genes associated with steroidogenesis, spermatogenesis, and gonadal development in the male chorus frogs within 6 h. Several gene pathways were similarly affected in the male chorus frogs receiving hormone injections to induce sperm development and release, implying there are parallels in hypothalamic-pituitary-gonadal axis stimulation induced by reception of conspecific cues and hormonal induction. We provide some of the first evidence of amphibian social signals causing rapid molecular changes linked to fertility regulation.

**Supplementary Information:**

The online version contains supplementary material available at 10.1038/s41598-026-43613-6.

## Introduction

Reproduction is a fundamental component of species ecology and evolution. Understanding social, environmental, and physiological drivers of reproduction is essential for explaining seasonal timing and reproductive success. In seasonally breeding species, external cues are transduced by the neuroendocrine system to stimulate physiological processes^1^. Reproductive endocrinology research focuses on the regulation of the hypothalamic-pituitary-gonadal (HPG) axis, production of gonadal sex steroids (i.e., androgens, estrogens), the expression of reproductive behaviours, and how internal and external factors impact these processes^2–4^. Considerable emphasis has been placed on how predictable environmental cues, such as temperature and photoperiod, signal the appropriate conditions for breeding, and how social interactions or secondary environmental cues then modulate neuroendocrine system to coordinate breeding to the correct time and context^2–4^. Direct links between reception of social cues and downstream gonadal effects, however, remain limited^5^, and is the focus of our study.

Anurans (toads and frogs) are amenable models for this question because acoustic communication coordinates reproduction by enabling mate localization and reproductive synchrony. Vocalizations (“calls”) are almost exclusively produced by males and are the primary method by which individuals can identify conspecifics, defend territories, assess the quality of potential competitors, and attract mates^6^. Growing evidence in anurans indicates a bidirectional relationship between endocrine reproductive control and acoustic reception^7–9^. Gonadal steroids influence hearing by enhancing the sensitivities of the sensory neurons in both sexes, and acoustic signals modulate gonadal steroid hormone production, either increasing or decreasing production of androgens and estrogens^4,10^. In addition to gonadotropins, arginine vasotocin, and adrenal steroids, the gonadal steroids are required to maintain male calling behaviour^4,9^. In many species, conspecific calls can stimulate other males to produce calls, demonstrating sensory feedback that links acoustic processing, reproductive state, and vocal output^6^. Previous research shows that conspecific calls modulate immediate early gene expression in the torus semicircularis, an important auditory processing region in frogs^11^, and in cells in the pallidum involved in processing of sensory signals^12^. Whole-brain RNA sequencing in female frogs exposed to conspecific male visual and acoustic cues demonstrates strong upregulation of energy-metabolism transcriptional networks^13^. Gonadal gene expression responses to reproductive or social behaviours in frogs are currently uncharacterised. Chorusing increases circulating androgens^14,15^ and testes mass^16^ in male frogs, yet the molecular mechanisms driving these testicular effects are unresolved.

Through a series of laboratory experiments, we have investigated the effect of conspecific signals (“chorus”) on the calling behaviour, reproductive output, and testicular gene expression in the boreal chorus frog (*Pseudacris maculata*), an explosive breeder and diploid species in the Hylidae family. Field observations^17^ indicated that a few males begin calling in the first days of the breeding season, which attracts and invokes calling in many males, and the chorusing group attracts females soon thereafter. Males produce clear chorusing and advertisement calls in early spring with tight seasonal synchrony, facilitating the study of the effects of social cues on gonadal function. We report that playback of recorded conspecific calls increased egg viability and tadpole survival for breeding couples. Results from transcriptomic analysis of testes indicate that conspecific male calls rapidly increase gene expression pathways associated with steroidogenesis, spermatogenesis and fertility, providing a plausible mechanism underlying acoustic enhancement of reproduction in the chorus frog.

## Results

### Experiment 1

**Fig. 1:**
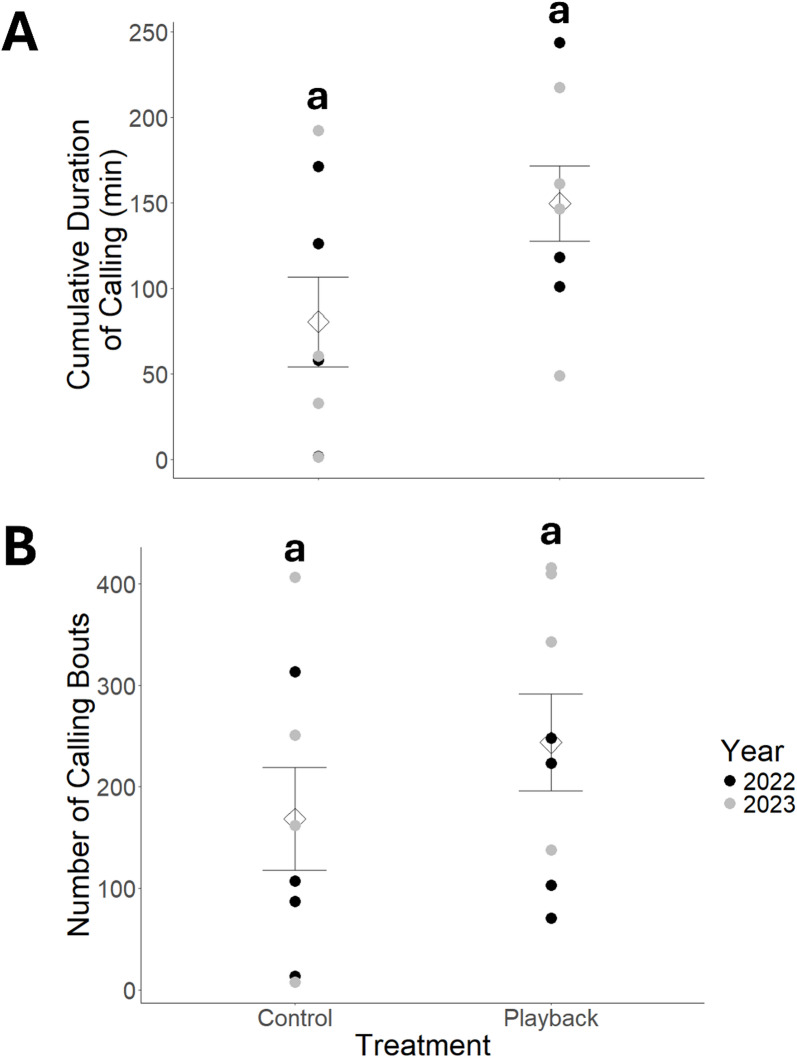
Comparison of calling behaviour of male boreal chorus frogs (*Pseudacris maculata*), including (**A**) mean cumulative duration and (**B**) mean number of calling bouts between two acoustic treatments. Control = control playback (April 20–26, 2022 and April 16–22, 2023) where the audio file contained ambient sounds (i.e., wind, rain, trees swaying). Playback = acoustic playback treatment (April 13–19, 2022 and April 23–30, 2023) where the audio file contained 10–12 chorus frogs calling. Individual circles correspond to the cumulative duration of calling and number of calling bouts per breeding container. Mean values per treatment are indicated by diamonds with standard error bars. Statistically significant differences (α = 0.05) are indicated by dissimilar lower-case letters.

We compared calling activity and reproductive output between groups of 10 boreal chorus frogs (4 females, 6 males) exposed to either a broadcast conspecific signals (“playback”) or a broadcast of natural sounds containing no calling individuals (“control”) during two breeding seasons. Calling effort was highly variable in both the control and playback groups (Fig. 1). The overall model was not significant for cumulative duration of calling (*F*_2,13_ = 2.01, *p* = 0.173, adjusted R^2^ = 0.119) nor the number of calling bouts (*F*_2,13_ = 2.46, *p* = 0.124, adjusted R^2^ = 0.163). However, the mean cumulative duration of calling was nearly two times (186%) longer in the playback group than the control with frogs calling for 149.7 min (± 62.3 min) and 80.6 min (± 74.1 min), respectively, despite the difference not being significant (*df* = 13, *t* = 1.96, *p* = 0.072). The mean cumulative duration of calling also did not differ between years (*df* = 13, *t* = -0.427, *p* = 0.676). There was a 145% increase in the number of calling bouts in the playback group (243.6 ± 135.4 bouts) compared to the control group (168.1 ± 143.6 bouts), but the difference was not statistically significant (*df* = 13, *t* = 1.18, *p* = 0.261). The number of calling bouts also did not differ between years (*df* = 13, *t* = 1.88, *p* = 0.083).

**Fig. 2:**
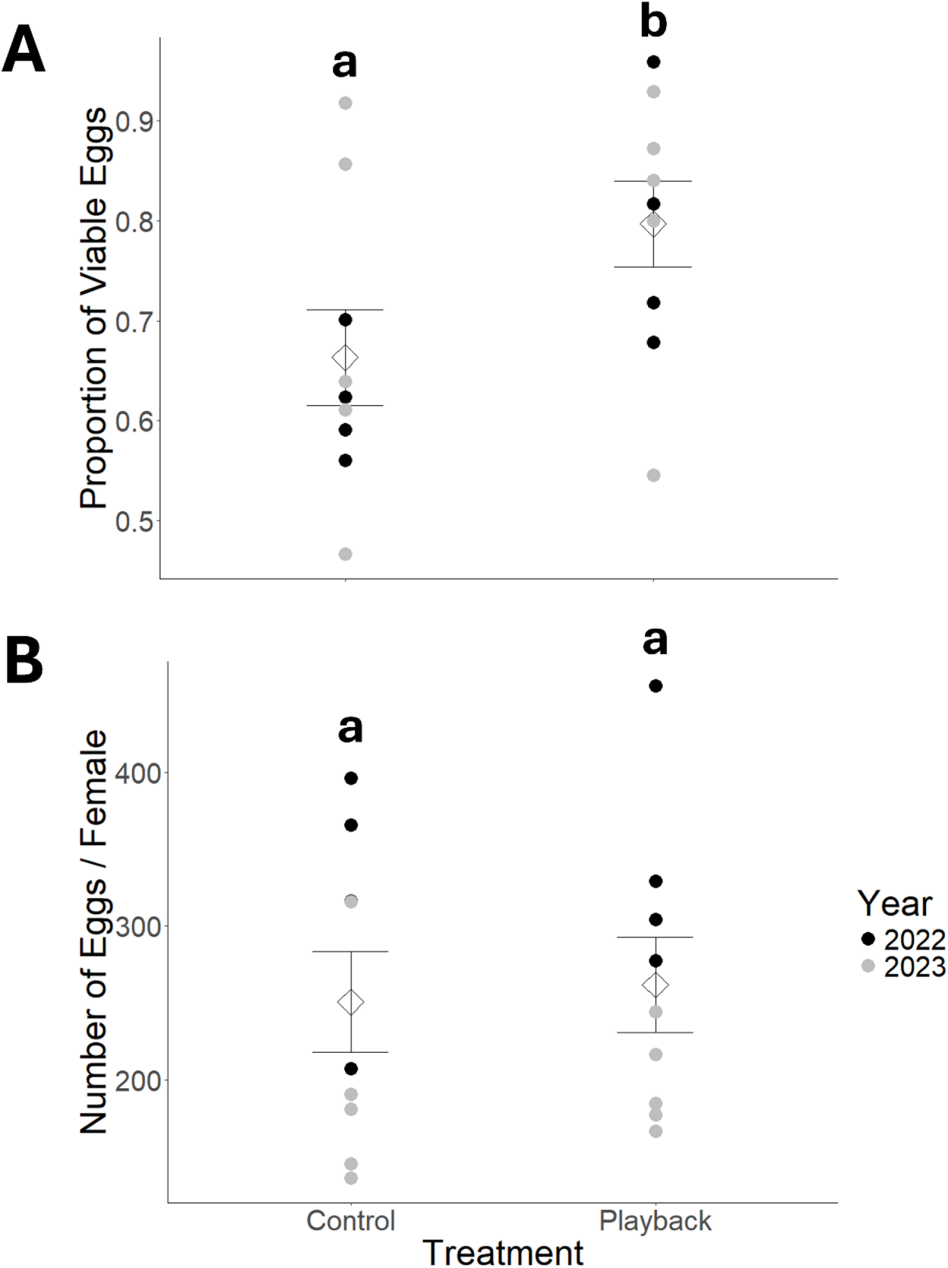
Comparison of reproductive output of spawning boreal chorus frogs (*Pseudacris maculata*) including (**A**) mean proportion of viable eggs and (**B**) mean number of eggs per female between two acoustic treatments. Control = control playback (April 20–26, 2022 and April 16–22, 2023) where the audio file contained ambient sounds (i.e., wind, rain, trees swaying). Playback = acoustic playback treatment (April 13–19, 2022 and April 23–30, 2023) where the audio file contained 10–12 chorus frogs calling. Individual circles correspond to the proportion of viable eggs per breeding container and number of eggs per female. Mean values per treatment are indicated by diamonds with standard error bars. Statistically significant differences (α = 0.05) are indicated by dissimilar lower-case letters.

Spawning rates ranged from 50 to 100% in both the control and playback groups. Furthermore, an equal number of females spawned in the control and playback groups (3.2 ± 0.7 females in both groups) and there was no significant difference in the spawning rate between years (*df* = 15, *t* = -0.15, *p* = 0.880). Viability was significantly higher in the playback group compared to the control group (*df* = 5, *z* = -2.13, *p* = 0.033). The mean proportion of viable eggs in the playback group was 0.797 (± 0.129) compared to 0.663 (± 0.143) in the control group (Fig. 2A), a 13.3% increase in egg viability (Cohen’s *d* = 0.978). There was no difference in the viability of eggs between years (*df* = 5, *z* = 0.79, *p* = 0.427). While the overall model was significant (*F*_2,15_ = 9.64, *p* = 0.002, adjusted R^2^ = 0.504) and there was a significant difference in the number of eggs produced between years (*df* = 15, *t* = -4.38, *p* < 0.001), acoustic playback did not affect the number of eggs per female (*df* = 15, *t* = 0.36, *p* = 0.722), with a mean of 250.7 (± 98.3) eggs per female in the control and 261.8 (± 92.7) eggs per female in the playback group (Fig. 2B).

**Fig. 3:**
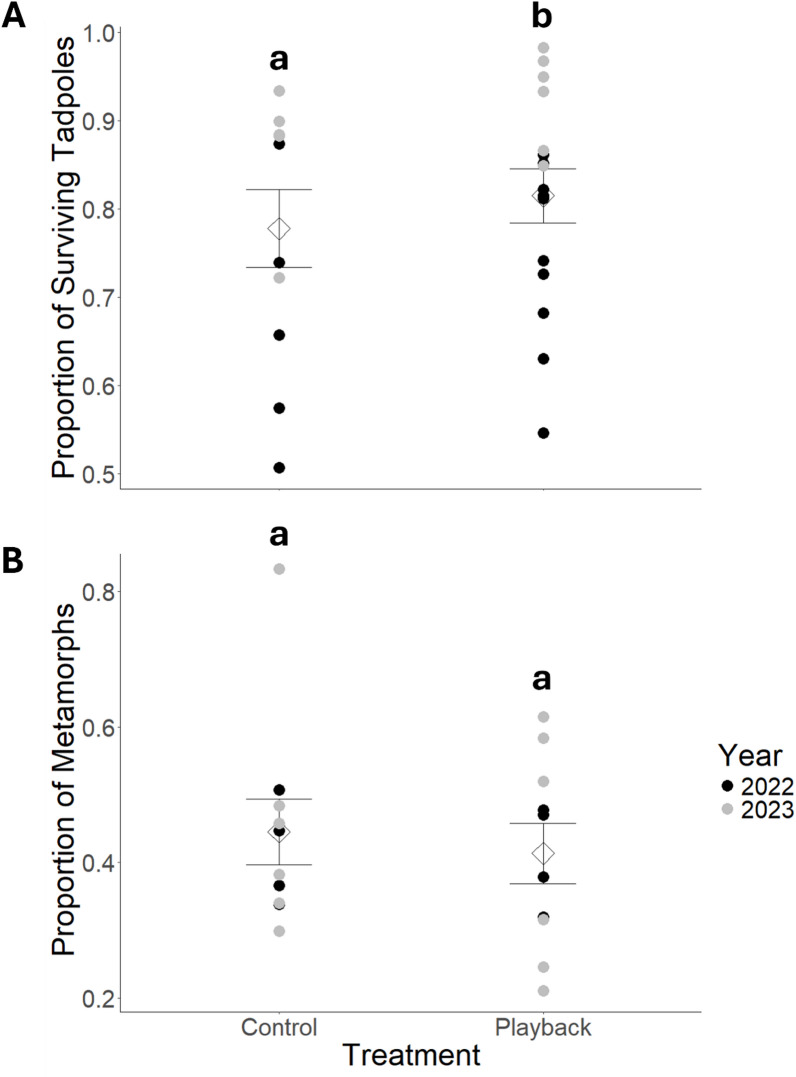
Comparison of tadpole quality of spawning boreal chorus frogs (*Pseudacris maculata*) including (**A**) mean proportion surviving tadpoles 46 days post-hatching and (**B**) mean proportion of tadpoles emerging as metamorphs (**B**) between two acoustic treatments. Control = control playback (April 20–26, 2022 and April 16–22, 2023) where the audio file contained ambient sounds (i.e., wind, rain, trees swaying). Playback = acoustic playback treatment (April 13–19, 2022 and April 23–30, 2023) where the audio file contained 10–12 chorus frogs calling. Individual circles correspond to the proportion surviving tadpoles 46 days post-hatching and proportion of tadpoles emerging as metamorphs per rearing tray. Mean values per treatment are indicated by diamonds with standard error bars. Statistically significant differences (α = 0.05) are indicated by dissimilar lower-case letters.

We compared the development and survival of tadpoles produced in breeding experiments. Tadpole survival (Fig. 3A) was significantly affected by treatment group (*df* = 5, *z* = 2.02, *p* = 0.043) and year (*df* = 5, *z* = 5.20, *p* < 0.001). While the mean proportion of surviving tadpoles was 0.815 (± 0.119) in the playback group compared to 0.778 (± 0.140) in the controls, the effect size was small (Cohen’s *d* = 0.279). When combining the control and playback groups, tadpole survival was 0.896 (± 0.066) in 2023 compared to 0.723 (± 0.115) in 2022 (Cohen’s *d* = 1.433). In contrast, metamorph emergence was not affected by treatment group (*df* = 5, *z* = -0.64, *p* = 0.525) or year (*df* = 5, *z* = -0.40, *p* = 0.690). The mean proportion of tadpoles emerging as metamorphs was 0.445 (± 0.145) and 0.414 (± 0.133) in the control and playback group, respectively (Fig. 3B). The overall model for mean length of the larval period was significant (*F*_2,17_ = 8.69, *p* = 0.002, adjusted *R*^2^ = 0.455). Length of the larval period was comparable between groups (*df* = 17, *t* = -0.31, *p* = 0.764) with a mean length of 97.4 (± 10.6) and 96.3 (± 10.6) days in the control group and playback group, respectively. However, length of the larval period was significantly different between years (*df* = 17, *t* = -4.22, *p* < 0.001), lasting 105.7 (± 4.4) days in 2022 compared to 91.0 (± 8.9) days in 2023 (Cohen’s *d* = 1.978).

### Experiment 2

Increased egg viability and tadpole survival for breeding couples exposed to playback led us to postulate that acoustic signals may increase gonadal function. Prolonged exposure to chorusing behaviour can increase circulating androgen concentration^14,15^ and testes mass^18^ in male frogs, but there have been no attempts to understand the mechanisms underlying testicular responses. Chorus size and calling intensity of conspecific males may also affect the gonadal function and hormonal state. We therefore compared the testicular gene expression profiles of male chorus frogs exposed to broadcasts of one of three intensities of conspecific calls: a small chorus of approximately 10–12 males (“Low Chorus”), a large chorus of approximately 100 males (“High Chorus”) or a recording of the natural breeding environment with no calling males (“Wind”). We also compared testicular gene expression profiles of male frogs injected with either a hormone mixture that effectively induces spawning (Amphiplex method^19,20^ or saline vehicle (Saline). We expected that gene expression patterns should be similar among frogs injected with the hormone exposure and frogs exposed to broadcasts of conspecific signals, reflecting stimulation of the HPG axis.

**Fig. 4:**
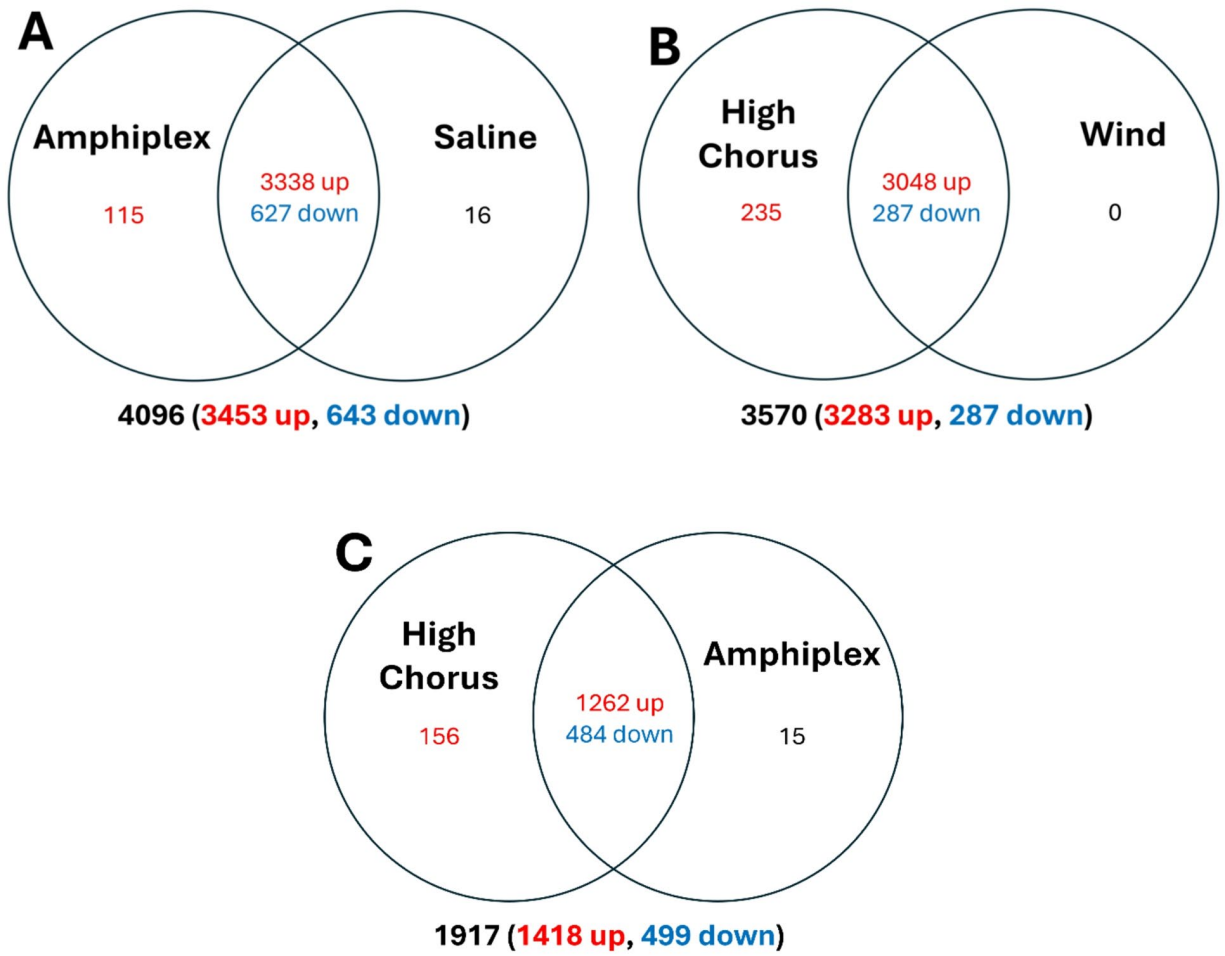
Venn diagrams of shared and unique differential expressed genes (DEGs) related to testis samples of boreal chorus frogs (*Pseudacris maculata*). In the first comparison (**A**) male chorus frogs were injected with a hormone mixture (“Amphiplex”) or 7% saline solution (“Saline”). In the second comparison (**B**) chorus frogs were exposed to 6-h broadcasts of a large chorus of 100 conspecific males calling (“High Chorus”) or natural sounds recorded within the breeding environment lacking frog calls (“Wind”). In the third comparison (**C**), males exposed to the High Chorus treatment are compared to the males that received the Amphiplex treatment. Values below Venn diagrams represent the total number of DEGs upregulated (red) and down regulated (blue) in each comparison.

The testicular transcriptome analysis identified 25,727 transcripts that were annotated in at least one of the six databases, representing 13,988 genes. Within the Amphiplex x Saline comparison, 1605 and 483 annotated transcripts were unique to the Amphiplex and Saline treatments, respectively. Within the High Chorus x Low Chorus x Wind comparison, there were 3979, 97, and 374 annotated transcripts unique to the High Chorus, Low Chorus, and Wind treatments, respectively. There were 4096 significant differentially expressed genes (DEGs) in the Amphiplex x Saline comparison, including 3453 upregulated, 643 downregulated genes (Fig. 4A). When comparing Low Chorus to Wind, we observed that there were only 17 DEGs (Supplementary Material; Table S1). Inspection of the z-scores among High Chorus, Low Chorus, and Wind treatments confirm that gene expression was similar between Low Chorus and Wind treatments. There were 3570 significant DEGs (3283 upregulated, 287 downregulated) in the High Chorus x Wind comparison (Fig. 4B). In the High Chorus x Amphiplex comparison, there were 1917 significant DEGs (1418 upregulated, 499 downregulated), with 156 genes being expressed in the High Chorus only (Fig. 4C).

Within the Amphiplex x Saline comparison, 2178 GO terms and 147 KEGG pathways were enhanced in the enrichment analyses. The DEGs in the Amphiplex treatment were associated with GO terms “male gonad development” (i.e., *NASP*, nuclear autoantigenic sperm protein; *INSL3*; insulin like 3), “cellular response to gonadotropin-releasing hormone” (i.e., *GNRHR2*, gonadotropin releasing hormone receptor-2; *ANXA5*, annexin A5), “cellular response to hormone stimulus” (i.e., signal transducer and activator of transcription isoforms; transcription factor isoforms; serine/threonine-protein kinases), and “steroid hormone binding” (i.e., sodium/potassium-transporting ATPase subunits). KEGG pathways significantly enriched by DEGs in the Amphiplex treatment that were potentially associated with reproduction and calling behaviour included “ovarian steroidogenesis” (*STAR*, steroidogenic acute regulatory protein; *PRKACA*, protein kinase cAMP-activated catalytic subunit alpha), a pathway that includes genes also important for testicular steroidogenesis and androgen signalling.

Within the High Chorus x Wind comparison, 2142 GO terms and 174 KEGG pathways were enhanced in the enrichment analyses. Notably, the High Chorus treatment and the Amphiplex treatment had several enhanced GO terms in common that were associated with spermatogenesis and response to steroid hormones (Table 1). The High Chorus treatment also had significantly enhanced genes associated with GO terms “male gonad development” (i.e., *NASP*, nuclear autoantigenic sperm protein; *INSL3*; insulin like 3), “spermatoproteasome complex” (i.e., proteasome subunits), “spermatogenesis” (i.e., spermatogenesis-associated proteins; testis-specific serine/threonine-protein kinases), and “follicle-stimulating hormone signaling pathway” (i.e., *ADRM1*, 26 S proteasome ubiquitin receptor; *ARRB2*, arrestin beta 2; *GRK2*, G protein-coupled receptor kinase 2). Many of the genes associated with spermatogenesis GO term were also upregulated in the High Chorus x Amphiplex comparison, including spermatogenesis associated proteins (*Spata19*, *Spata16*, *Spata20*), testis-specific transition proteins (*Tnp1*, *Tnp2*), and histone H1 or H1-like proteins found in spermatids (*Hils1*, *H1fnt*). KEGG pathways significantly enriched by DEGs in the High Chorus treatment included “prolactin signaling pathway” (*CYP17A1*, cytochrome P450 family 17 subfamily A member 1; prolactin precursors; prolactin-like proteins), “oxytocin signaling pathway” (serine/threonine-protein phosphatases; myosin light polypeptides), “cortisol synthesis and secretion” (cAMP responsive element binding protein 3-like proteins; cyclic AMP-dependent transcription factors), and “estrogen signaling pathway” (*MMP*, matrix metalloproteinase-2; type I keratins; heat shock proteins).

**Table 1:** List of enhanced GO pathways related to spermatogenesis and hormone secretion observed in both the Amphiplex x Saline and the High Chorus x Wind comparisons.

GO Term	Genes in pathway	Amphiplex x Saline				High Chorus x Wind			
		DE genes - total	DE genes - up	DE genes - down	*p*-value	DE genes - total	DE genes - up	DE genes - down	*p*-value
response to hormone	32	16	15	1	**< 0.01**	14	14	0	**< 0.01**
hormone activity	73	22	20	2	**< 0.01**	25	22	3	**< 0.01**
cellular response to peptide hormone stimulus	15	7	7	0	**< 0.01**	8	7	1	**< 0.01**
steroid biosynthetic process	34	16	14	2	**< 0.01**	14	14	0	**< 0.01**
spermatid development	104	9	8	1	0.98	41	34	7	**< 0.01**
binding of sperm to zona pellucida	68	16	10	6	**0.04**	42	41	1	**< 0.01**
Spermatogenesis	383	61	55	6	0.30	163	150	13	**< 0.01**
male gonad development	66	16	15	1	**0.03**	25	24	1	**< 0.01**
steroid metabolic process	38	11	11	0	**0.02**	14	14	0	**0.01**
response to steroid hormone	21	5	4	1	**0.01**	8	8	0	**0.04**

We then described how gene expression changed between three time points (6, 24, and 30 h post-exposure) in the Low Chorus and High Chorus broadcast groups by examining the expression pattern of the genes within the GO terms “steroid biosynthetic process”, “response to hormone”, and “male gonad development” (Supplementary Material, Table S2). Most of the genes within the GO terms that were upregulated in the High Chorus at time point 1 (6 h) remained significantly upregulated at time point 2 (24 h) and at time point 3 (30 h) when compared to the Wind at the same timepoints (Table 2). However, many genes that were upregulated at time point 1 were significantly downregulated at time point 2 when comparing within the High Chorus, and there were little to no significant DEGs between time point 2 and time point 3. This pattern was repeatedly observed even when considering GO terms that contained several dozen or hundreds of genes. For example, for the “spermatogenesis” term, 119/163 (73%) genes were significantly upregulated (log2FC > 1.5, *p* < 0.05) in the High Chorus treatment compared to the Wind. Gene expression in the Low Chorus between time points differed from the High Chorus. Very few or no DEGs were found in the Low Chorus x Wind at time point 1 and time point 3, but several genes associated with GO terms “steroid biosynthetic process”, “response to hormone”, and “male gonad development” were upregulated at time point 2. Additionally, the expression of genes in the Low Chorus x Wind comparison at time point 2 was similar to the expression of genes in the High Chorus x Wind comparison at time point 1 (Table 3).

**Table 2:** Summary of testicular gene expression within High Chorus treatment in comparison to Wind treatment at three timepoints (6–30 h). Coloured nodes (red) indicate upregulation of gene based on log2 fold change (1.5 > log2FC < -1.5) and adjusted p-value (< 0.05).

Steroid biosynthetic process (GO:0006694)							
ID	Gene	High 6 h x		High 24 h x		High 30 h x	
		Wind 6 h		Wind 24 h		Wind 30 h	
		log2FC	*P*(adj)	log2FC	*P*(adj)	log2FC	*P*(adj)
TRINITY_DN111256_c1_g3	*Dhcr24*	**2.96**	0.009	0.85	0.982	**5.17**	0
TRINITY_DN113871_c0_g2	*FDFT1*	**2.53**	0.038	**2.11**	0.002	**3.96**	0
TRINITY_DN116674_c2_g3	*Tspo*	**5.48**	0	**5.24**	0	**7.45**	0
TRINITY_DN131188_c0_g1	*Scp2d1*	5.36	0.475	–	–	–	–
TRINITY_DN19878_c0_g1	*Cyp17a1*	**5.65**	0.002	2.19	1	2.5	1
TRINITY_DN54102_c0_g1	*Hsd17b11*	**5.01**	0	**4.58**	0.012	**5.77**	0
TRINITY_DN65460_c2_g2	*Hsd17b12*	**4.14**	0	2.84	0.2	**5.16**	0
TRINITY_DN74172_c4_g1	*Cyb5r1*	1.54	0.435	**2.36**	0.008	**3**	0
TRINITY_DN78062_c2_g2	*Sc5d*	**3.41**	0	**4.13**	0.027	**4.78**	0
TRINITY_DN78735_c0_g1	*Scp2*	**4.11**	0	**2.22**	0.032	**4.06**	0
TRINITY_DN83670_c0_g1	*Cyb5r3*	**4.6**	0	**3.57**	0.029	**4.93**	0
TRINITY_DN90946_c0_g2	*Tecr*	**4.21**	0	3.03	0.084	**5.24**	0
TRINITY_DN91956_c2_g1	*Hmgcs1*	**3.57**	0	1.97	0.326	**5.72**	0
TRINITY_DN95069_c3_g1	*Msmo1*	**2.82**	0.025	1.98	0.242	**3.69**	0

Response to hormone (GO:0009725)							
ID	Gene	High 6 h x		High 24 h x		High 30 h x	
		Wind 6 h		Wind 24 h		Wind 30 h	
		log2FC	*P*(adj)	log2FC	*P*(adj)	log2FC	*P*(adj)
TRINITY_DN110262_c3_g5	*Timp2*	2.07	0.278	2.51	0.067	**3.53**	0.005
TRINITY_DN118595_c0_g1	*Mmp14*	1.49	0.535	**2.84**	0.005	**3.9**	0
TRINITY_DN118956_c0_g3	*Lox*	2.26	0.235	2.87	0.152	3.53	0.505
TRINITY_DN130422_c0_g1	*Mb*	**6.49**	0	**5.34**	0.043	6.37	0.187
TRINITY_DN130843_c0_g1	*GPX1*	0.5	1	–	–	–	–
TRINITY_DN64833_c0_g2	*Cox5b*	**5.33**	0	3.38	0.079	**5.97**	0
TRINITY_DN65559_c0_g11	*Mtnd3*	**6.6**	0	2.14	0.876	**4.45**	0.002
TRINITY_DN68843_c3_g1	*HCLS1*	**4.61**	0	**2.62**	0.025	**2.65**	0.002
TRINITY_DN76207_c0_g1	*SORD*	**3.49**	0.008	1.8	0.082	**3.84**	0
TRINITY_DN80017_c0_g1	*NCOA4*	**4.94**	0	**3.6**	0.011	**6.83**	0
TRINITY_DN84281_c3_g2	*Timp1*	2.66	0.075	**3.48**	0.004	**4.72**	0
TRINITY_DN84747_c0_g1	*Me1*	**3.96**	0	**3.48**	0.002	**6.17**	0
TRINITY_DN87262_c0_g4	*Aqp1*	**6.78**	0	1.59	0.866	**4.06**	0.046
TRINITY_DN97922_c2_g1	*Por*	**3.97**	0	**2.31**	0.007	**4.15**	0

Male gonad development (GO:0008584)							
ID	Gene	High 6 h x		High 24 h x		High 30 h x	
		Wind 6 h		Wind 24 h		Wind 30 h	
		log2FC	*P*(adj)	log2FC	*P*(adj)	log2FC	*P*(adj)
TRINITY_DN100710_c0_g3	*Insl6*	**5.53**	0.018	1.04	1	2.06	1
TRINITY_DN106049_c0_g2	*BCL2L1*	**3.01**	0.001	**3.18**	0	**5.06**	0
TRINITY_DN106744_c1_g1	*Cited2*	**3.26**	0.006	**3.84**	0	**6.41**	0
TRINITY_DN107686_c0_g1	*Ybx3*	**5.16**	0	1.8	0.355	**3.88**	0
TRINITY_DN109406_c2_g1	*Kdr*	**5.4**	0	1.86	0.892	**4.69**	0.017
TRINITY_DN109503_c2_g2	*Eif2s2*	**2.66**	0.032	**2.48**	0.008	**3.88**	0
TRINITY_DN112537_c3_g2	*Tbc1d20*	**3.56**	0	1.95	0.09	**2.63**	0.002
TRINITY_DN112800_c0_g1	*Wdr48*	**4.82**	0	2.25	0.081	**5.74**	0
TRINITY_DN115851_c1_g4	*PRPS1*	3.91	0.635	–	–	–	–
TRINITY_DN123522_c0_g1	*CSDE1*	**2.38**	0.007	**2.06**	0.022	**4.01**	0
TRINITY_DN130557_c0_g1	*Klhl10*	**21.56**	0	–	–	–	–
TRINITY_DN1311_c0_g1	*ANKRD7*	4.93	0.543	–	–	–	–
TRINITY_DN131297_c0_g1	*Spink2*	5.95	0.38	–	–	–	–
TRINITY_DN56774_c0_g1	*Nupr1*	1.77	0.297	2.72	0.206	**6.17**	0
TRINITY_DN64913_c0_g2	*Rbp4*	**7.05**	0	**6.08**	0.002	**5.86**	0
TRINITY_DN66237_c2_g2	*Insl3*	**7.19**	0.002	-0.04	1	1.77	1
TRINITY_DN69790_c0_g1	*Prdx4*	2.31	0.09	1.73	0.453	**4.07**	0
TRINITY_DN69790_c0_g3	*PRDX4*	5.5	0.453	–	–	–	–
TRINITY_DN73303_c4_g4	*Fdps*	2.53	0.079	**4.64**	0.001	**4.14**	0
TRINITY_DN74726_c4_g1	*Bax*	**2.68**	0.006	**3.71**	0	**4.92**	0
TRINITY_DN86395_c1_g2	*Hmgb2*	2.59	0.073	1.44	0.404	**4.22**	0
TRINITY_DN86973_c0_g1	*SFRP2*	2.46	0.158	**5.78**	0	2.39	0.66
TRINITY_DN94714_c3_g4	*Six4*	-0.3	0.22	-0.07	1	-0.12	1
TRINITY_DN95226_c4_g4	*CITED2*	**3.77**	0	**3.06**	0	**5.18**	0
TRINITY_DN96193_c2_g1	*NASP*	1.96	0.419	**2.43**	0	**2.29**	0.028

**Table 3:** Summary of gene expression within High Chorus and Low Chorus treatments in comparison to Wind treatment. Coloured nodes (red) indicate upregulation of gene based on log2 fold change (1.5 > log2FC < -1.5) and adjusted p-value (< 0.05).

Steroid biosynthetic process (GO:0006694)							
ID	Gene	Low 6 h x		High 6 h x		Low 24 h x	
		Wind 6 h		Wind 6 h		Wind 24 h	
		log2FC	*P*(adj)	log2FC	*P*(adj)	log2FC	*P*(adj)
TRINITY_DN111256_c1_g3	*Dhcr24*	-0.06	0.999	**2.96**	0.009	2.82	0.182
TRINITY_DN113871_c0_g2	*FDFT1*	-1.87	0.76	**2.53**	0.038	**3.42**	0
TRINITY_DN116674_c2_g3	*Tspo*	-0.33	1	**5.48**	0	**6.21**	0.003
TRINITY_DN131188_c0_g1	*Scp2d1*	–	–	5.36	0.475	–	–
TRINITY_DN19878_c0_g1	*Cyp17a1*	-0.56	1	**5.65**	0.002	0.3	1
TRINITY_DN54102_c0_g1	*Hsd17b11*	-1.57	1	**5.01**	0	**6.99**	0
TRINITY_DN65460_c2_g2	*Hsd17b12*	-0.47	0.998	**4.14**	0	3.95	0.08
TRINITY_DN74172_c4_g1	*Cyb5r1*	-3.58	0.356	1.54	0.435	**4.75**	0
TRINITY_DN78062_c2_g2	*Sc5d*	-3.01	1	**3.41**	0	**5.81**	0.001
TRINITY_DN78735_c0_g1	*Scp2*	-0.4	0.998	**4.11**	0	**2.98**	0.032
TRINITY_DN83670_c0_g1	*Cyb5r3*	-1.86	0.942	**4.6**	0	**4.62**	0.03
TRINITY_DN90946_c0_g2	*Tecr*	-0.09	0.998	**4.21**	0	**4.34**	0.012
TRINITY_DN91956_c2_g1	*Hmgcs1*	-1.22	0.977	**3.57**	0	**3.64**	0.007
TRINITY_DN95069_c3_g1	*Msmo1*	-1.89	0.846	**2.82**	0.025	**3.62**	0.005

Response to hormone (GO:0009725)							
ID	Gene	Low 6 h x		High 6 h x		Low 24 h x	
		Wind 6 h		Wind 6 h		Wind 24 h	
		log2FC	*P*(adj)	log2FC	*P*(adj)	log2FC	*P*(adj)
TRINITY_DN110262_c3_g5	*Timp2*	-2.32	0.854	2.07	0.278	**5.19**	0
TRINITY_DN118595_c0_g1	*Mmp14*	-2.23	0.769	1.49	0.535	**4.17**	0
TRINITY_DN118956_c0_g3	*Lox*	-4.15	0.648	2.26	0.235	**4.87**	0.032
TRINITY_DN130422_c0_g1	*Mb*	2.93	1	**6.49**	0	-0.06	1
TRINITY_DN130843_c0_g1	*GPX1*	–	–	0.5	1	1.02	1
TRINITY_DN64833_c0_g2	*Cox5b*	0.03	1	**5.33**	0	4.12	0.091
TRINITY_DN65559_c0_g11	*Mtnd3*	1.72	0.914	**6.6**	0	1.95	0.877
TRINITY_DN68843_c3_g1	*HCLS1*	-0.03	0.999	**4.61**	0	-0.86	0.94
TRINITY_DN76207_c0_g1	*SORD*	-1.55	0.902	**3.49**	0.008	**3.91**	0.001
TRINITY_DN80017_c0_g1	*NCOA4*	-1.05	0.993	**4.94**	0	**5.06**	0.008
TRINITY_DN84281_c3_g2	*Timp1*	-5.57	0.142	2.66	0.075	**5.29**	0
TRINITY_DN84747_c0_g1	*Me1*	-0.39	0.998	**3.96**	0	**4.13**	0.001
TRINITY_DN87262_c0_g4	*Aqp1*	4.04	1	**6.78**	0	-2.74	1
TRINITY_DN97922_c2_g1	*Por*	-0.83	0.956	**3.97**	0	1.81	0.125

Male gonad development (GO:0008584)							
ID	Gene	Low 6 h x		High 6 h x		Low 24 h x	
		Wind 6 h		Wind 6 h		Wind 24 h	
		log2FC	*P*(adj)	log2FC	*P*(adj)	log2FC	*P*(adj)
TRINITY_DN100710_c0_g3	*Insl6*	–	–	**5.53**	0.018	3.43	1
TRINITY_DN106049_c0_g2	*BCL2L1*	-2.38	0.658	**3.01**	0.001	**4.47**	0
TRINITY_DN106744_c1_g1	*Cited2*	-1.65	0.91	**3.26**	0.006	**4.09**	0
TRINITY_DN107686_c0_g1	*Ybx3*	0.66	0.995	**5.16**	0	2.79	0.106
TRINITY_DN109406_c2_g1	*Kdr*	-0.58	1	**5.4**	0	2.67	0.643
TRINITY_DN109503_c2_g2	*Eif2s2*	-1.38	0.925	**2.66**	0.032	**3.78**	0
TRINITY_DN112537_c3_g2	*Tbc1d20*	-1.69	0.799	**3.56**	0	**4.8**	0
TRINITY_DN112800_c0_g1	*Wdr48*	-2.22	1	**4.82**	0	**3.38**	0.011
TRINITY_DN115851_c1_g4	*PRPS1*	–	–	3.91	0.635	–	–
TRINITY_DN123522_c0_g1	*CSDE1*	-1.43	0.836	**2.38**	0.007	**3.16**	0.001
TRINITY_DN130557_c0_g1	*Klhl10*	–	–	**21.56**	0	–	–
TRINITY_DN1311_c0_g1	*ANKRD7*	–	–	4.93	0.543	–	–
TRINITY_DN131297_c0_g1	*Spink2*	–	–	5.95	0.38	–	–
TRINITY_DN56774_c0_g1	*Nupr1*	-2.27	0.885	1.77	0.297	**4.48**	0.013
TRINITY_DN64913_c0_g2	*Rbp4*	0.52		**7.05**	0	2.59	1
TRINITY_DN66237_c2_g2	*Insl3*	–	–	**7.19**	0.002	0.3	1
TRINITY_DN69790_c0_g1	*Prdx4*	-2.11	0.759	2.31	0.09	**3.38**	0.013
TRINITY_DN69790_c0_g3	*PRDX4*	–	–	5.5	0.453	–	–
TRINITY_DN73303_c4_g4	*Fdps*	-2.09	0.924	2.53	0.079	**6.55**	0
TRINITY_DN74726_c4_g1	*Bax*	-2.03	0.699	**2.68**	0.006	**5.41**	0
TRINITY_DN86395_c1_g2	*Hmgb2*	-2.18	0.72	**2.59**	0.073	**3.02**	0.011
TRINITY_DN86973_c0_g1	*SFRP2*	-3.09	1	2.46	0.158	**3.88**	0.008
TRINITY_DN94714_c3_g4	*Six4*	0.27	0.72	-0.3	0.22	-0.09	0.962
TRINITY_DN95226_c4_g4	*CITED2*	-1.09	1	**3.77**	0	**3.38**	0.001
TRINITY_DN96193_c2_g1	*NASP*	-2.31	0.708	**1.96**	0.419	**3.08**	0.009

## Discussion

We provide empirical evidence that broadcasts of male conspecific calls result in stimulation of testicular function in male boreal chorus frogs. Prolonged exposure to broadcasts of chorus behaviour increased egg viability in spawning pairs of boreal chorus frogs and increased the survival rate of tadpoles several weeks post-hatching. To our knowledge, this is the first evidence in an amphibian that social signals can cause rapid molecular changes in the gonads (i.e., differential gene expression). We found that playback of male choruses increased the expression of genes associated with steroidogenesis, spermatogenesis, and gonadal development in male chorus frogs within 6 h. We also observed increased male calling activity after prolonged exposure to playbacks observed during spawning, implying that there are positive feedback relationships among reception of calls, testicular activity, and call production in boreal chorus frogs.

We also report for the first time an increase in egg viability and tadpole survival in anurans in response to an acoustic playback during spawning. However, there is some evidence that broadcasts of conspecific calls can increase reproductive investment in birds. Playback of either their mate’s song or recordings from conspecific colonies led to zebra finches (*Taeniopygia guttata*) laying larger clutches compared to the absence of an acoustic treatment^21^. Similarly, female canaries (*Serinus canaria*) exposed to male conspecific calls produce more eggs compared to those that heard no songs and playbacks of synthetic sounds mimicking highly attractive mates resulted in larger eggs being produced^22^. Additionally, in captive brown-headed cowbirds (*Molothrus ater*) the number of eggs produced per day was significantly correlated with the number of counter-singing interactions among males^23^.

Despite the current lack of evidence that social cues can enhance fertility in amphibians, there is strong evidence that the regions of the brain associated with acoustic reception have neural projections to nuclei that regulate reproduction in vertebrates^7–9,24,25^. Neurons in auditory regions, such as the torus semicircularis, project to hypothalamic nuclei and regulate reproductive endocrine outputs^4,7,8^. Acoustic cues are transduced by the preoptic area and ventral hypothalamus, which leads to activation of gonadotropin-releasing hormone (GnRH) neurons^7,10^. GnRH release into the median eminence-portal system triggers luteinizing hormone (LH) and follicle-stimulating hormone (FSH) secretion from pituitary gonadotrophs. These pituitary hormones then stimulate oogenesis, spermatogenesis, and steroid hormone synthesis^11–13^. For example, after hearing conspecific calls for several consecutive days, male green treefrogs (*Dryophytes cinereus*) exhibited a significant 25% increase in the number of immunoreactive GnRH neurons accompanied by increased plasma androgen production^24^. Similarly, male grass frogs (*Rana temporaria*) maintain mature testes (i.e., increased testis volume and interstitial tissue size) longer when exposed to a synthetic signal that resembled conspecific calls but were unaffected by a synthetic signal that resembled heterospecific calls^18^. In female midwife toads (*Alytes muletensis*), eggs will continue to mature when exposed to conspecific male calls but are reabsorbed if conspecific calls cease or when exposed to heterospecific calls^26^. The relationship between sex steroid hormone concentrations and gamete quality/quantity is well established in anuran amphibians^27–29^ and fish^30,31^. Increases in circulating concentrations of androgens and estrogens induced by exposure to biologically relevant social cues likely maintain higher quality and quantity of gametes which then increase the likelihood of successful fertilization of eggs.

The gene expression analysis of our study further supports our hypothesis that social cues can stimulate reproduction by activating endocrine pathways that upregulate genes involved in gonadal function and fertility regulation. There was considerable overlap in the groups of genes that were differentially expressed in Amphiplex x Saline and High Chorus x Wind comparisons for the testicular samples, including several GO terms, indicating shared hormonal response to the co-administration of GnRH and metoclopramide and the reception of conspecific male calls. GO terms “response to hormone”, “male gonad development”, and “steroid biosynthetic process” were significantly upregulated in both Amphiplex x Saline and High Chorus x Wind comparisons.

There were several notable individual genes within KEGG pathways that were upregulated in High Chorus and Amphiplex males, including *Insl3*, *Klhl10*, *Cyp17a1*, *Tspo*, *Hsd17b11*, *Hsd17b12*, and *Aqp1*. Insulin-like peptide 3 (INSL3) has a role in spermatogenesis and germ cell survival, stimulating the differentiation of spermatogonia in zebrafish^32^. INSL3 is also a biomarker for testicular function, with its concentration reflecting differentiation status and number of the Leydig cells present in the testes^33,34^. Kelch-like family member 10 (Klhl10) is exclusively produced in testes within developing spermatids and knockout of *Klhl10* allele led to the disruption of spermiogenesis and complete male infertility in mice^35,36^. Cytochrome P450 family 17 subfamily A member 1 (CYP17A1) is an important enzyme in synthesis of glucocorticoids and sex hormones, particularly for the conversion of pregnenolone (P5) to 17-hydroxypregnenolone and progesterone (P4) to 17-hydroxyprogesterone is 17α-hydroxylase activity and production of androgens via 17,20-lyase activity^37^. CYP17A1 also has a role in sexual behaviour and secondary sexual characteristics in males. *Cyp17a1* knockout leads to infertility and absence of sexual behaviours in mice^38^. In frogs, *Cyp17a1* is highly expressed in the gonads before and after sexual differentiation in males and likely important for testis development^39,40^. Translocator protein (TSPO) is abundant in steroidogenic cells, including Leydig cells, and important for the transport of cholesterol into mitochondria to initiate steroid hormone synthesis^41,42^. The functions of 17 beta-hydroxysteroid dehydrogenases (e.g., Hsd17b11, Hsd17b12) have been inferred for amphibians as all vertebrates appear to express some form of the enzyme^43^. *Hsd17b12* is highly expressed in meiotic and post-meiotic germ cells in mice and rats^44^ and in Leydig cells of mice^45^. In mice, aquaporin (Aqp1) is mainly involved in regulation of water homeostasis in male reproductive organs (i.e., testis, efferent ducts, seminal vesicles), which is essential for reproductive health by maintaining proper ionic conditions for maturation and storage of spermatozoa^46,47^.

Our results may indicate that a minimal chorus size, or threshold amount of calling, is necessary to initiate and sustain sufficient calling effort at breeding locations. Gene expression comparisons between Low Chorus and Wind were significantly different at the second time point (24 h) and several genes that were upregulated at 24 h in the Low Chorus x Wind comparison were also upregulated in the High Chorus x Wind comparison at 6 h. These results suggest that similar pathways or groups of genes associated with steroidogenesis and spermatogenesis are being activated in the Low Chorus and High Chorus groups, but it takes longer to occur in the Low Chorus. We suspect that the Low Chorus broadcast met the proposed minimal size/intensity requirement but does not far exceed it, resulting in the delayed response in gene activation in comparison to the High Chorus.

## Conclusion

Despite broad acceptance that social information has profound effects on reproduction, there is relatively little data on the influence of conspecific signals on HPG axis at the molecular level. Our gene expression results provide evidence for the reciprocal interaction between endocrine control of reproduction and the reception of conspecific acoustic signals^9^. We have identified several genes and gene pathways in male frogs that could be used as targets for studies on amphibian reproduction and for comparisons of hormonal activation of reproduction among vertebrate taxa. Further research is needed to determine the influence of calling parameters on gonadal gene expression, such as manipulations to calling rate, dominant frequency, sound pressure level of conspecific signals which have been shown to elicit behaviour responses^7,9^. Several aspects of courtship and mate characteristics that may influence fertilization success and egg viability were not captured in this study but could be considered in future investigations. Amplexus duration and position, male-to-female body size ratio, and age can affect fertilization success in some anurans^48,49^. Genetic compatibility may also influence fertilization success, early survival, and hatching success^50^. Studies such as ours on gonadal transcriptomics would also be strengthened by incorporating plasma or tissue hormone measurements (i.e., E2, T, CORT, LH/FSH) to further validate HPG axis activation.

## Methods

### Animal collection

Wild boreal chorus frogs were collected during the early breeding season (late March-early April) from ponds in the United Counties of Leeds and Grenville, Ontario, Canada (44.904440, -75.831694). Boreal chorus frogs are a small-bodied (mean snout-to-vent length of adults: 27 mm ♂, 30 mm ♀) frog species in the family Hylidae (tree frogs) that is native to Canada and the United States^51^. Boreal chorus frogs are a cold- and freeze-tolerant species, emerging from hibernation and beginning to spawn in late winter or early spring in a variety of ephemeral wetlands^51^. At the beginning of the breeding season, males gather in large groups, up to hundreds of individuals, and remain within the breeding habitat for 4–10 weeks. Conversely, females are present in the breeding habitat for less than 2 weeks. Sex ratios on breeding grounds are generally highly biased towards males^52^.

Male and female frogs were held separately in groups of 8 individuals in 1.7-L plastic terraria (18.3 × 8.5 × 10.8 cm) with damp moss and fed pinhead crickets every two days. To determine if female frogs were gravid, we used a combination of body shape and ultrasound imagining (Fujifilm Vevo F2 LT with UHF71x transducer probe; bandwidth: 71 − 30 MHz, scan depth: 10 mm). Additionally, if females weighed less than 0.80 g, they were not used in the experiments as they were likely immature or non-gravid. All males with conspicuous yellow-to-brown colouration of the vocal sac were assumed to be sexually mature.

### Ethics

For this study, wild frogs were collected from nature and held in captivity 7–14 days prior to the start of experimentation. Frog collection was conducted as per the Wildlife Scientific Collector’s Authorization (Permit # 1105089) issued by the Ontario Ministry of Natural Resources. In the first experiment, 90 adult frogs were used (54 males and 36 females). In the second experiment, 88 adult male frogs were used. All experiments were performed in accordance with relevant guidelines and regulations and were approved by the Canadian Council on Animal Care and the University of Ottawa Animal Care and Veterinary Service. The authors complied with the ARRIVE guidelines.

### Experiment 1

Frogs were placed into two groups; a treatment group (“playback”) where chorus frogs are exposed to a broadcast of conspecific males calling and a group (“control”) where chorus frogs are exposed to a playback of ambient sound recorded at a natural breeding pond. We chose a control recording of ambient pond sounds (i.e., wind, rain, crickets, trees swaying) to ensure that the observed responses were due to the broadcast of conspecific calls rather than those elicited by the reception of any source of sound. The spawning experiments were conducted over four 7-day trial periods with stimuli broadcasted for 6 h from 18:00–24:00 each day (Supplementary Material; Figure S1). To avoid any possibility of cross-communication, the experiments were conducted sequentially rather than simultaneously. In 2022, the playback exposure was performed before the control with the order reversed in 2023. The playback experiments conducted from April 13–19 in 2022 (*n* = 4) and April 23–29 in 2023 (*n* = 5), and the control trials conducted from April 20–26 in 2022 (*n* = 4) and April 16–22 in 2023 (*n* = 5).

Previous work with several frog species, including the boreal chorus frog, has indicated that spawning in a captive setting is improved by administering a low dose of a gonadotropin-releasing hormone (GnRH) agonist 24-hrs prior to the main hormone injection^19,20^. On Day 1, frogs were given an injection of 0.04 µg/g body weight of an GnRH agonist (des-Gly10, D-Ala6, Pro-LHRH; Bachem H4070.0005; GnRHa) in a 10 µL saline vehicle with a disposable 31-gauge needle attached to a 0.3 mL syringe. Twenty-four hours later, on Day 2, frogs received an injection of a hormone mixture (AMPHIPLEX method) containing the GnRHa (0.4 µg/g body weight) and a type 2 dopamine receptor antagonist, metoclopramide (Sigma; 10 µg/g body weight; MET)^19^. All injections were given between 16:00–18:00. In between Day 1 and Day 2 injections, male and female frogs were held separately in small 750 mL containers placed in a cooler with ice (4–6 °C).

After Day 2 injections, four female and six male frogs were placed in 114-L breeding container (Sterilite; 43.8 cm H x 82.9 cm L x 50.2 cm W) filled with 100 L of dechlorinated water. Breeding containers were placed 50 cm from each other. The frogs were provided with green garden fencing weighed down with rocks as an oviposition substrate. To simulate fluctuations in temperature in natural breeding pools, we allowed water temperature to vary throughout the day (Supplementary Material; Figure S2). Every morning at 9:00 from Day 3–7, breeding containers were surveyed for the general health of frogs, the number of couples in amplexus, and the number of egg clusters laid.

A 6-hr composite audio tape of approximately 10–12 male chorus frogs calling was used as the treatment playback. The audio tape was produced using recordings of chorus frogs calling in the field during the breeding season (April–May) with a direction shotgun microphone (RODE NTG4) attached to a ZOOM H6 portable multi-track audio recorder^17^. To simulate the rising and falling in calling intensity, the audio file began with a calling rate of 43 calls/min for 1 h, increased to 68 calls/min for 4 h and then decreased to 43 calls/min for 1 h. For the control, a 6-hr audio file containing ambient pond sounds (i.e., wind, rain, trees swaying) from one of the frog collection sites recorded just prior to boreal chorus frog calling activity was used. Both the playback and control audio files were normalized in Audacity (version 3.0.2) to -1.0 dB. The audio files were broadcasted at 85 dB (at 1 m) daily for five days (Day 2–6) from 18:00 until 24:00 from speakers placed an equal distance (80 cm) from each breeding container. On Day 7, frogs were removed from the breeding containers, and the eggs were collected. The eggs were transferred to rearing trays (14.1 cm H x 38.3 cm L x 24.1 cm W) filled with 6 L of dechlorinated water held at 17–19 °C.

We recorded the vocalizations of male chorus frogs to estimate calling effort using a condenser microphone (APEX 185B) placed 15 cm above the center of the lid of each of the breeding containers. We connected microphones to a central multichannel A/D converter interface device (Behringer UMC404HD). As estimates of calling effort, the mean cumulative duration of calls and the mean number of calling bouts were determined in the playback and control groups. Cumulative duration was defined as the total sum of the duration in minutes of all calls produced within a breeding container within the first 12 h. A calling bout was defined as a series of calls produced with less than 2 s between calls. Water and air temperatures were recorded every 10 min using waterproof temperature loggers (Onset HOBO 8 K Pendant).

Egg numbers were determined by photographing trays from above and manually counted by two observers. The average number of eggs of the counts was rounded to the nearest whole integer. Eggs per female were calculated by dividing the number of number of eggs in a breeding container by the number of spawning females in a breeding container. To estimate fertilization rate, we determined the proportion of viable eggs four days post-oviposition. Egg viability was estimated by photographing the trays from above (40 cm from base of tray to camera lens). At four days post-oviposition, viable and non-viable eggs can be easily differentiated from each other as tadpoles begin to develop (Supplementary Material; Figure S3). Photographs of eggs were divided into 32 cells of equal area, and the number of viable and non-viable eggs was counted in five of the cells selected randomly using a random number generator. The proportion of viable eggs was therefore the number of viable eggs in the five cells divided by the total number of eggs counted in the five cells. Spawning was confirmed at the end of each experimental trial. After humane sacrifice by overdose of tricaine methanesulfonate (Syndel USA Syncaine, Catalog No. NC0872873), the spinal cord of females was transected, and the body cavity opened and visually inspected for the presence of eggs or evidence of spawning.

To estimate the quality of offspring produced in each treatment, we determined the mean proportion of tadpoles that survived to approximately 46 days post-hatching, the mean proportion of tadpoles reaching metamorphosis, and the length of the larval period. The post-hatch age of tadpoles was approximate as hatching occurs 5–7 days post-oviposition and it is extremely difficult to track individuals. Two weeks after hatching, tadpoles from the control group and playback group were separated on May 11 in 2022 (Control: 5 replicates, Playback:10 replicates) and May 18 in 2023 (6 replicates per group) and reared at densities of 6–10 tadpoles/L. Gosner stage (Gs)^53^ of the tadpoles ranged from Gs 27–Gs 31 and tadpoles of different stages equally divided between groups. Every other day between 10:00 h–14:00 h tadpoles were fed mixture of phytoplankton, algae, boiled spinach, and frog brittle (Supplementary Material; Table S3). Three times a week, excess food was removed and 80% of the water was replaced to prevent water fouling. Mean proportion of tadpole survival was based on the number of tadpoles survived until June 15 in 2022 and June 20 in 2023. We chose mid-June (46 days post-hatching) because chorus frog tadpoles begin to reach metamorphic climax starting in early July, thus approximating survival during the larval period^52^. Mean proportion of tadpoles reaching metamorphosis was the proportion of tadpoles reaching metamorphic climax (Gs 42) from May 11 in 2022 (4 replicates per group) and May 18 in 2023 (6 replicates per group) until September 01 in both years (approx. four months). Length of larval period was the mean number of days elapsed between the hatch date and date of metamorphic climax. Both environmental (i.e., food availability, space) and neuroendocrinological factors affect time to metamorphosis^54^, and not all tadpoles will metamorphose. Since environmental factors such food, water quality, and space were comparable between groups and years, the proportion of tadpoles that emerge as metamorphs and the length of the larval period approximates the differences in development affected by the playback treatment.

All statistical analyses were performed in R (Version 4.3.3). A series of models was fitted with treatment and year as independent variables. First, linear regression models were used to compare the mean number of eggs per female, mean calling bouts, mean cumulative duration of calling, and mean length of the larval period. Assumptions of linear models were evaluated using regression diagnostic plots generated with the base R plot function. Second, beta regression models were applied to test differences in the mean proportion of egg viability, proportion of surviving tadpoles, and proportion of emerging metamorphs between years and groups using the betareg function in the betareg package^55^. Binomial regression models weighted by the total number of eggs or tadpoles were initially explored, but variance inflation factor tests for dispersion (c_hat function in the AICcmodavg package)^56^ indicated overdispersion and lack of fit (c-hat > 4). Beta regression is appropriate for modelling continuous data bounded between 0 and 1 and is more flexible than the more traditionally used binomial regression model^57^. No p-value adjustment for multiple comparisons (e.g., Bonferroni correction) was applied, to balance the risks of Type I (false positive) and Type II (false negative) errors; instead, effect sizes (Cohen’s *d*) are reported for all significant comparisons^58^. Effect sizes are defined as low, medium, and large for Cohen’s d ≥ 0.2, 0.5, and 0.80, respectively^58,59^. All descriptive statistics are expressed as mean values (± standard deviation) unless stated otherwise.

### Experiment 2

The evening before the first day of the experiment, groups of 6–8 frogs were placed in small containers (946 mL; 14 cm H x 11 cm W) and were allowed to gradually warm overnight in a portable cooler to ambient room temperature (e.g., 18 °C). At 8:30 the following morning, frogs were transferred to 114-L containers (Sterilite; 43.8 cm H x 82.9 cm L x 50.2 cm W) with 100 L of dechlorinated water and plastic platforms for perching. Frogs were allowed to acclimate to the conditions of the room (i.e., light, temperature, disturbance from being moved) for 1.5 h. Containers were a standard distance of 1 m from the playback speaker. At 10:00, one of three treatments (“Wind”, “Low Chorus”, “High Chorus”) was broadcasted at sound pressure level of 85 dB detected at 1 m. The first container was removed at 16:00 (Timepoint 1 = 6 h), the second was removed at 10:00 the next day (Timepoint 2 = 24 h) and the third was removed at 16:00 of the second day (Timepoint 3 = 30 h). The order of treatments in each year was randomized and then ran consecutively to minimize time difference between treatments and thus time that the frogs were held before they were exposed.

During the short, intensive breeding season, boreal chorus frogs will call throughout the 24-hr period, during both the day and the night. Calling activity is most intense during the late afternoon/early evening and steeply declines after midnight (Supplementary Material; Figure S4). The Low Chorus treatment simulated a small population of 10–12 chorus frogs, which was adapted from the Playback treatment used in Experiment 1. The low chorus broadcast began at 10:00 with 2 h of silence until 12:00, followed by 2 h of two males calling from 12:00 to 14:00, then 7 h of ten males calling from 14:00 to 21:00, and then 7 h of two males calling from 21:00 to 4:00 the next morning. From 4:00 to 10:00 there were 6 h of silence. The High Chorus treatment simulated a large population of 80–100 chorus frogs. The broadcast began at 10:00 with 2 h of two males calling until 12:00, followed by 2 h of ten males calling from 12:00 to 14:00, then 7 h of approximately 80–100 males calling from 14:00 to 21:00, and then 7 h of ten males calling from 21:00 to 4:00 the next morning. From 4:00 to 10:00 there are 6 h of two males calling. The Wind treatment simulated natural sounds (i.e., wind, rain, insects, and trees swaying) that were recorded at breeding locations prior to the breeding season and therefore do not contain calling behaviour of any frog or toad species. The Wind treatment is the same 6-h audio recording used in the Control treatment of Experiment 1, which was repeated four and five times for Timepoint 2 (24 h) and Timepoint 3 (30 h), respectively.

After broadcasts, frogs were removed from the 114-L containers, humanely euthanized with an overdose of buffered 0.5% tricaine solution (Syndel Syncaine, Thermo Fisher Scientific, Waltham, MA, USA), and spinal cord was cut. The testes were removed for RNA extraction. As a positive control, we investigated gonadal gene expression profiles in response to a hormonal treatment following a gonadotropin-releasing hormone (GnRH) priming protocol (“Amphiplex”). Following the injection procedure used in experiment 1, we intraperitoneally injected a group of 8 male chorus frogs with a low priming dose of a GnRH agonist (GnRH-A; 0.04 µg/g body weight des-Gly10, D-Ala6, Pro-LHRH; Bachem H4070.0005) followed 24 h later with an injection of a mixture of GnRH-A (0.4 µg/g) and the type 2 dopamine receptor antagonist metoclopramide (10 µg/g) ^19^. In the negative control treatment (“Saline”), we injected frogs with a 0.7% saline solution.

After dissection, testis samples (*n* = 40) were stored in RNA later^®^ (Thermo Fisher Scientific, Waltham, MA, USA), kept at 4 °C for 24 h, and then frozen to -80 °C. Upon thawing, the samples were homogenized using a VWR 4-Place Mini Bead Mill Homogenizer (VWR^®^, Atlanta, GA, USA), and total RNA was purified from the homogenates using NucleoZOL reagents with NucleoSpin RNA columns (Macherey-Nagel, Germany). RNA concentrations were measured with a Qubit 4 Fluorometer (Invitrogen™, Thermo Fisher Scientific, Waltham, MA, USA), and RNA integrity (RIN) was assessed using an Agilent 4150 TapeStation system (Agilent Technologies, Inc., Santa Clara, CA, USA). All samples were of high quality (mean RIN = 9.8) and yield (mean concentration = 0.057 µg/µL). rPoly(A) RNA sequencing library was performed by LC Sciences Inc. (https://lcsciences.com, Houston, TX, USA) and was prepared following Illumina’s TruSeq-stranded-mRNA sample preparation protocol. Paired-ended sequencing was performed on Illumina’s NovaSeq 6000 sequencing system.

As there is no current annotated genome or transcriptome for the boreal chorus frog, a *de novo* assembly was produced by LC Sciences. Cutadapt^60^ and custom Perl scripts (https://www.perl.org/about.html) were used to remove the reads that contained adaptor contamination, low quality bases and undetermined bases. Sequence quality was then verified using FastQC (http://www.bioinformatics.babraham.ac.uk/projects/fastqc). All downstream analyses were based on this clean data of high quality. *De novo* assembly of the transcriptome was performed with Trinity 2.4.0^61^. Trinity groups transcripts into clusters, loosely defined as a ‘gene’, based on shared sequence content. Statistical significance was adjusted for multiple comparisons using the false discovery rate (FDR) correction^62^.

All assembled genes were aligned against the National Center for Biotechnology Information non-redundant (Nr) protein database (http://www.ncbi.nlm.nih.gov), Gene ontology (GO) (http://www.geneontology.org), SwissProt (http://www.expasy.ch/sprot), Kyoto Encyclopedia of Genes and Genomes (KEGG) (http://www.kegg.jp/kegg)^63^ and eggNOG (http://eggnogdb.embl.de) databases using the DIAMOND algorithm^64^ with a threshold of E-value < 0.00001. Differential expression analysis of genes was performed using Salmon^65^ based on normalized transcript per million (TPM) values^66^. The differentially expressed genes (DEGs) were selected as the transcripts that were annotated in at least one of the six databases with a log2 fold change of > 1 or <-1 and with statistical significance (FDR p-value < 0.05) using the R package “edgeR”^67^. Enrichment analysis was performed, mapping DEGs to GO terms (https://www.geneontology.org/docs/ontology-documentation) and KEGG pathways (https://www.genome.jp/kegg/mapper/search.html), with significance based on the hypergeometric equation:

$$P=1-\sum_{i=0}^{S-1}\frac{\left(\begin{array}{c}B\\i\end{array}\right)\left(\begin{array}{c}TB-B\\TS-i\end{array}\right)}{\left(\begin{array}{c}TB\\TS\end{array}\right)}$$

where TB gene number = number of total genes in the comparison; TS gene number = number of differentially expressed genes in total genes in the comparison; B gene number = total number of genes in the GO term/KEGG pathway; S gene number = number of differentially expressed genes in the GO term/KEGG pathway. GO terms/KEGG pathways with p-value < 0.05 were considered significantly enhanced. Significantly enhanced genes within terms/pathways are noted in parentheses in the Results section. Note that the naming of genes, GO terms, and KEGG pathways are largely based on previous genomic and transcriptomic research in mice and humans.

## Supplementary Information

Below is the link to the electronic supplementary material.

Supplementary Material 1

## Data Availability

The data discussed in this publication have been deposited in NCBI’s Gene Expression Omnibus^68^ and are accessible through GEO Series accession number GSE315762 (https://www.ncbi.nlm.nih.gov/geo/query/acc.cgi?acc=GSE315762).
