# Supplementary material for: Reception of conspecific cues alters testicular gene expression and improves fertility in boreal chorus frogs (Pseudacris maculata)
Source: Sci Rep. 2026 Mar 11;16:13073. doi: 10.1038/s41598-026-43613-6 (PMC13099952; doi:10.1038/s41598-026-43613-6)
Supplement: Supplementary file 1 — Supplementary Material 1 [file 41598_2026_43613_MOESM1_ESM.docx]

**Supplementary Materials**

**Reception of conspecific cues alter testicular gene expression and improve fertility in boreal chorus frogs (*Pseudacris maculata*)**

Ethier JP, Lee H, Robinson SA, Trudeau VL

**
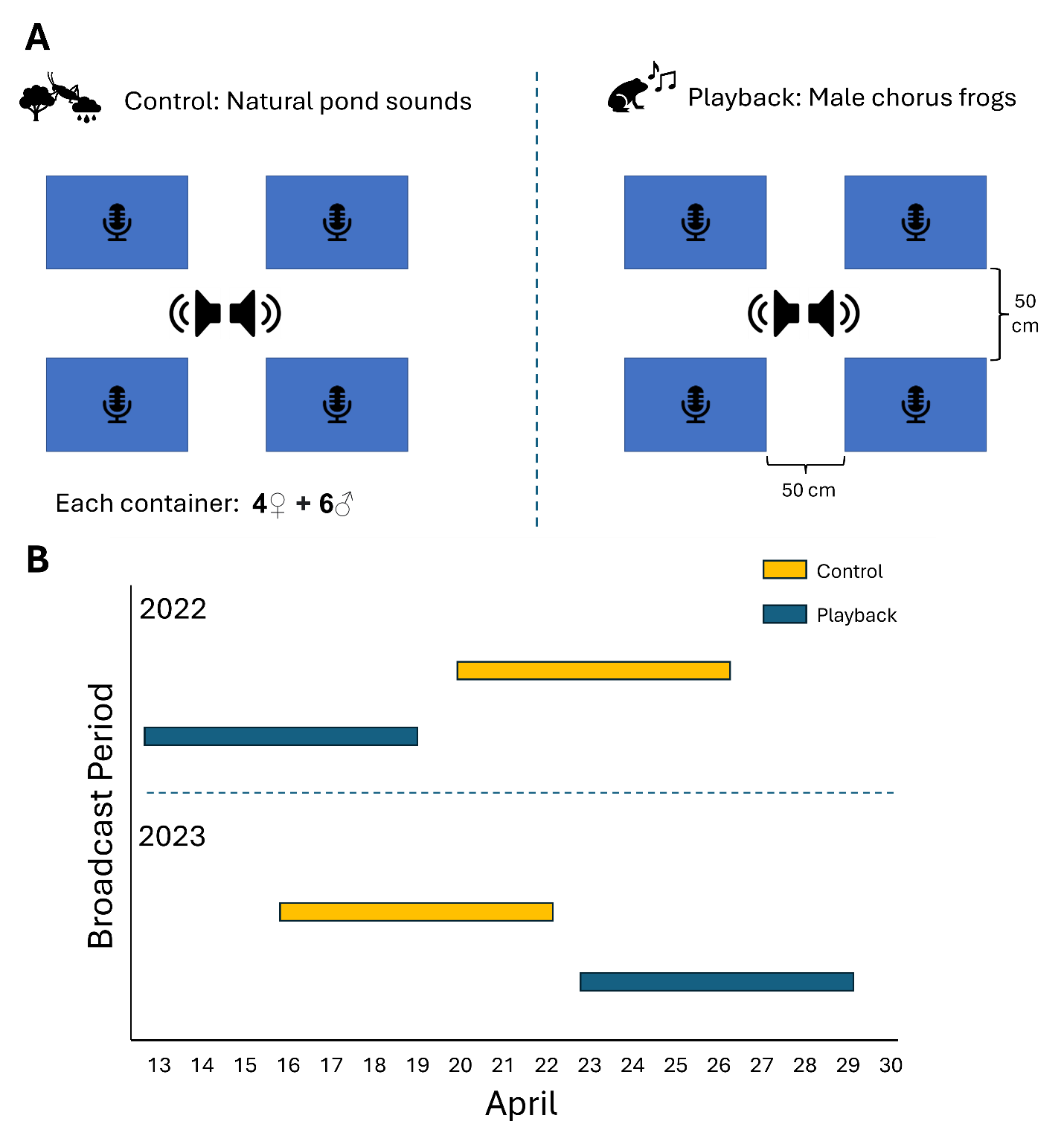
Figure S1**: (**A**) The setup of boreal chorus frog (*Pseudacris maculata*) spawning experiment. In each breeding container (blue boxes), four female and six male frogs are placed with 100 L of dechlorinated water and green garden fencing to use as an oviposition substrate. Breeding containers were spaced 50 cm apart from each other. A condenser microphone (APEX 185B) was placed 15 cm above the lid of each of the breeding containers, which are connected to a central multichannel A/D converter interface device attached to a laptop to record vocalizations. In the middle of the breeding containers were two speakers, so the distance from the microphone on top of each breeding container to the speaker was 80 cm. (**B**) In the control group of frogs (April 20–26, 2022 and April 16–22, 2023) were exposed for 6 hr from 18:00–24:00 each day for five days to ambient sounds from a local breeding pond just prior to chorus frog breeding season (April–May) and prior to any calling behaviour. The playback group of frogs (April 13–19, 2022 and April 23–29, 2023) were exposed to a recording of conspecific frogs calling for 6 hr from 18:00–24:00 each day for five days. Note that the playback exposure was performed before the control in 2022 and then the order was reversed in 2023.

**Figure S2**: Water temperature (°C) of breeding containers during boreal chorus frog (*Pseudacris maculata*) spawning experiment. In the control group (April 20–26, 2022 and April 23–30, 2023) male and female frogs were exposed for 6 hr from 18:00–24:00 each day to ambient sounds from a local breeding pond recorded just prior to chorus frog breeding season (April–May) and prior to any calling behaviour of any frog species. In the playback group (April 13–19, 2022 and April 23–30, 2023) male and female frogs were exposed to a recording of a chorus of conspecific frogs calling for 6 hr from 18:00–24:00 each day. Day 1 frogs were injected with a priming dose of GnRHa (0.04 μg/g body weight) and then held at 4°C in a cooler. On Day 2 frogs were injected with a dose of Amphiplex hormone mixture (0.4 μg/g body weight GnRHa and 10 0.4 μg/g body weight MET) and placed into the breeding containers. Broadcasts of control or playback audio occurred during the evenings of Day 2 to Day 6. Frogs were removed from breeding containers on morning of Day 7.

**
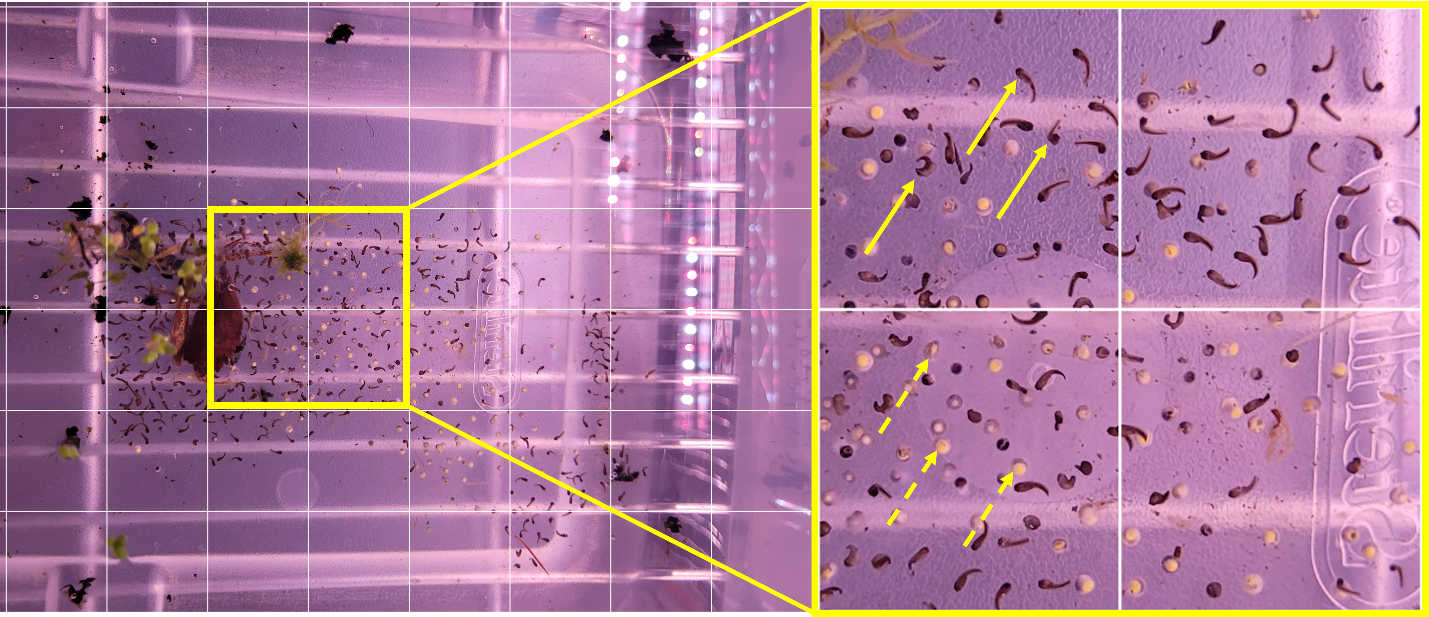
**

**Figure S3**: Example of the image analysis to determine the viability of boreal chorus frog (*Pseudacris maculata*) eggs in an acoustic playback experiment. Egg trays were photographed four days after being laid from a fixed distance of 40 cm. Images were then overlayed with a 32-cell grid. All eggs and developing tadpoles were counted in five randomly selected cells. Viable eggs/embryos (solid arrows) black, irregular shaped, and begun to develop, having often reached Gosner stage 15–19. Non-viable eggs (dashed arrows) are circular and white-to-yellowish in appearance with no obvious development.

**Figure S4**: Comparison of natural calling behaviour of boreal chorus frogs (*Pseudacris maculata*) and simulated number of calling individuals. ***Primary Y-axis***: Dashed grey line; the call index follows the standard call index of the North American Amphibian Monitoring Program -- (0) absence of calling activity; (1) up to two or three unique individuals, with mostly non-overlapping calls, (2) overlapping calls but still allowing to distinguish individuals, and (3) full chorus consisting of overlapping calls with non-distinguishable individuals. ***Secondary Y-axis***: Solid yellow and blue lines; simulated number of calling individuals (logarithmic scale) -- (0) absence of calling activity; (2); two or three calling individuals with occasional but minimal overlap; (10) three or four individuals calling in the spectral foreground of recording with several individuals in the spectral background, approximately 10 individuals in total, with a lot of overlap of calls; (100) full chorus consisting of overlapping calls with non-distinguishable individuals, approximately 80–100 individuals in total.

**
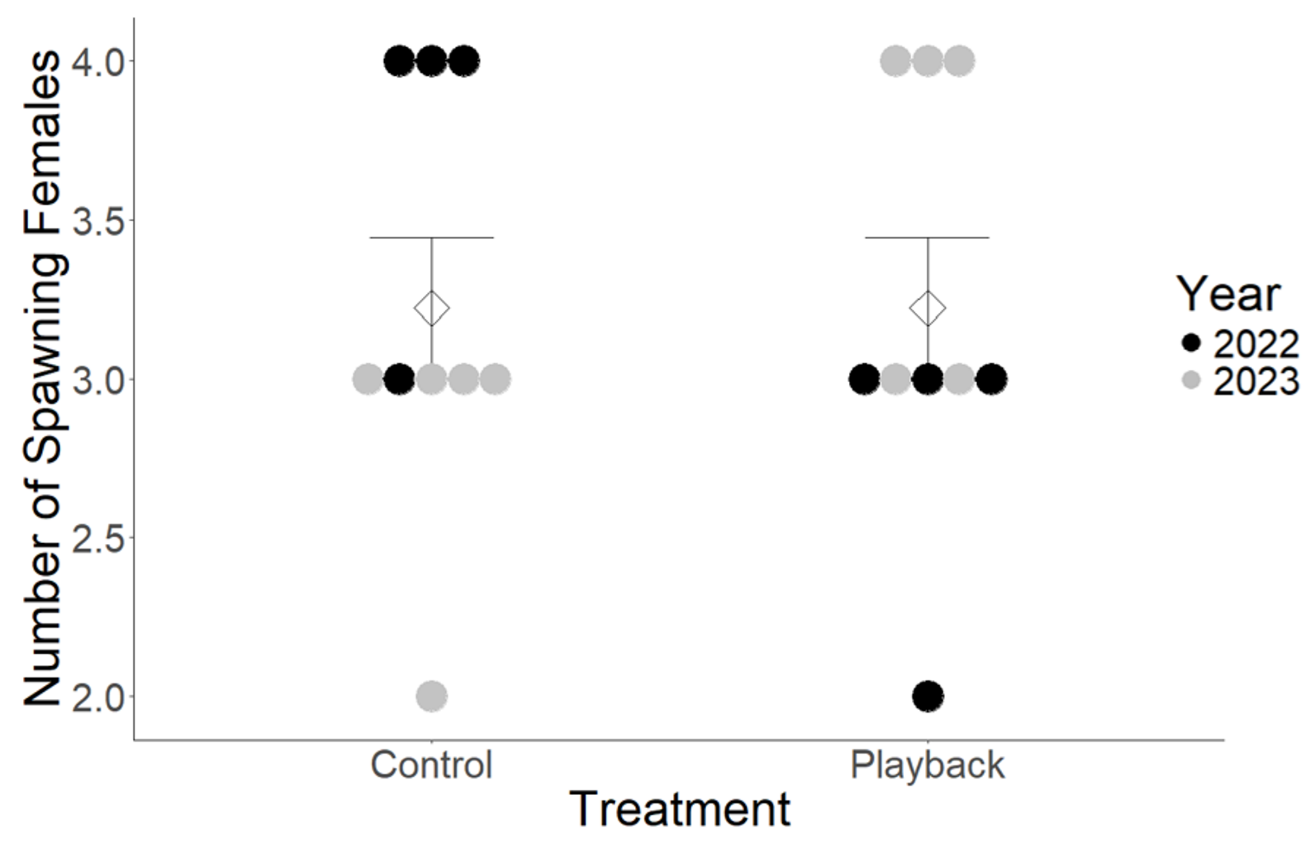
**

**Figure S5:** Comparison of the number of spawning female boreal chorus frogs (*Pseudacris maculata*) between two acoustic treatments. Control = control playback (April 20–26, 2022 and April 16–22, 2023) where the audio file contained ambient sounds (*i.e.*, wind, rain, trees swaying). Playback = acoustic playback treatment (April 13–19, 2022 and April 23–30, 2023) where the audio file contained 10–12 chorus frogs calling. Mean values are indicated by diamonds with standard error bars.

**
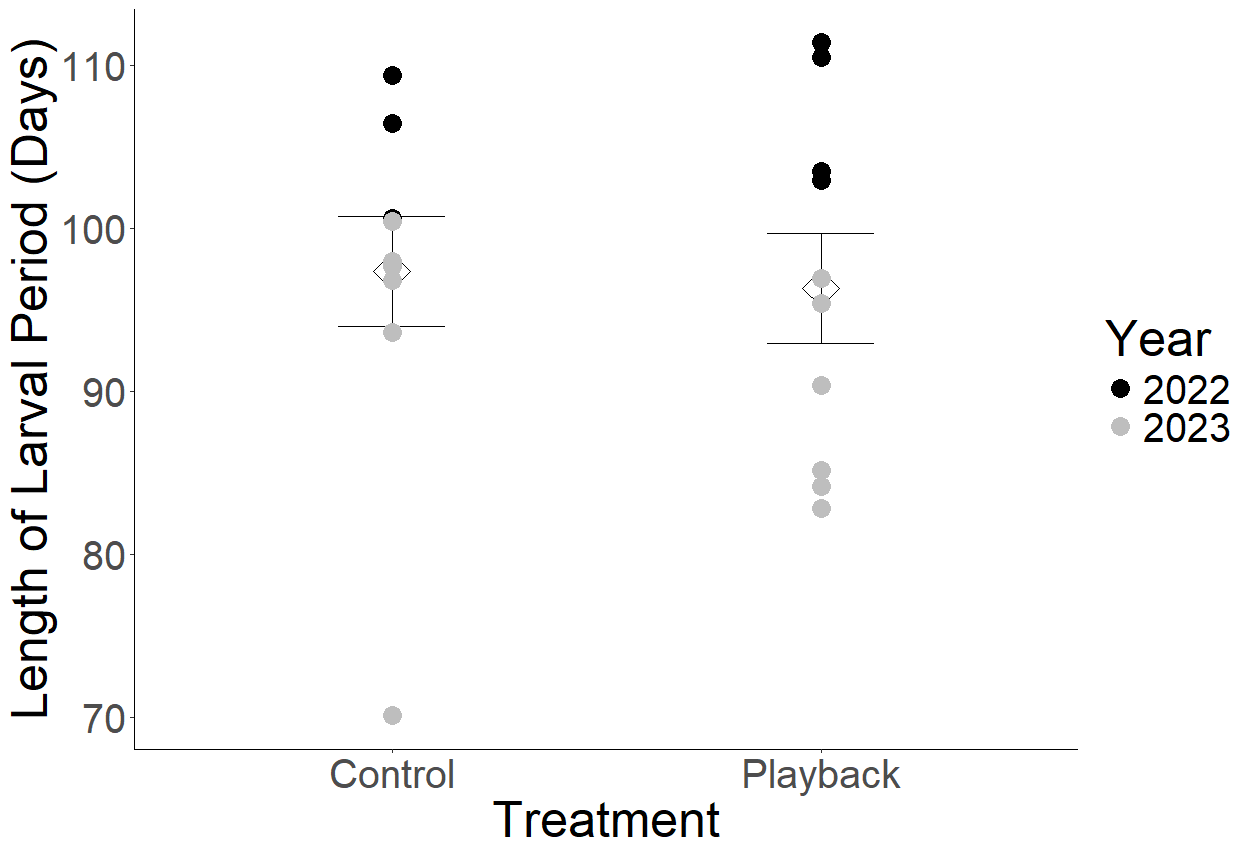
**

**Figure S6:** Comparison of the mean length of the larval period of tadpoles from spawning boreal chorus frogs (*Pseudacris maculata*) exposed to two acoustic treatments. Control = control playback (April 20–26, 2022 and April 16–22, 2023) where the audio file contained ambient sounds *(i.e.*, wind, rain, trees swaying). Playback = acoustic playback treatment (April 13–19, 2022 and April 23–30, 2023) where the audio file contained 10–12 chorus frogs calling. Mean values between treatments are indicated by diamonds with standard error bars.

**Table S1:** Differentially expressed genes in the Low Chorus x Wind comparison of testicular samples. Gene symbol and name based on description within the Swiss-Prot database (https://www.sib.swiss/swiss-prot).

| **Gene Symbol** | **Name**  **(species)** | **log2FC** | **P(adj)** |
| --- | --- | --- | --- |
| *Tnnt3* | troponin T3  (*Mus musculus*) | 7.14 | < 0.0001 |
| *METTL11B* | methyltransferase 1B  (*Xenopus tropicalis*) | -5.25 | 0.0027 |
| *ccnf* | cyclin-F  (*Nanorana parkeri*) | 5.25 | 0.0027 |
| *Rab3il1* | guanine nucleotide exchange factor for Rab-3A isoform X4 (*Nanorana parkeri*) | -5.17 | 0.0028 |
| *Pde4dip* | phosphodiesterase 4D interacting protein  (*Xenopus tropicalis*) | 5.17 | 0.0028 |
| *AOX2* | aldehyde oxidase-like  (*Nanorana parkeri*) | -5.04 | 0.0047 |
| *RRNAD1* | ribosomal RNA adenine dimethylase domain containing 1 (*Xenopus tropicalis*) | -4.76 | 0.0105 |
| *TMEM8C* | transmembrane protein 8C  (*Xenopus laevis*) | 4.72 | 0.0109 |
| *RGS19* | regulator of G-protein signaling 19 S homeolog isoform X2 (*Xenopus laevis*) | 4.64 | 0.0137 |
| *cat* | catalase  (*Nanorana parkeri*) | -4.55 | 0.0153 |
| *CRYAB* | Alpha-crystallin B chain  (*Lithobates catesbeiana*) | -4.53 | 0.0160 |
| *ADGRF3* | adhesion G-protein coupled receptor F3  (*Terrapene mexicana triunguis*) | -4.45 | 0.0205 |
| *Spp1* | osteopontin isoform 2  (*Mus musculus*) | -4.36 | 0.0296 |
| *Abcc8* | ATP-binding cassette sub-family C member 8-like (*Xenopus laevis*) | 4.33 | 0.0328 |
| *SLC34A2* | solute carrier family 34 member 2  (*Rana catesbeiana*) | -4.29 | 0.0385 |
| *CMAS* | N-acylneuraminate cytidylyltransferase  (*Nanorana parkeri*) | 4.21 | 0.0487 |
| *ZCCHC11* | zinc finger CCHC-type containing 11  (*Nanorana parkeri*) | 4.20 | 0.0494 |

Table S2: Summary of testicular gene expression at 6, 24, and 30 hrs within High Chorus and Low Chorus treatments. For each treatment the differential gene expression in comparison to the Wind treatment (6 hrs) is provided as reference. Colour indicates upregulation (red) or downregulation (blue) of gene based on log2 fold change (1.5 > log2FC < -1.5) and adjusted p-value (< 0.05).

| **steroid biosynthetic process (GO:0006694)** | | | | | |  |  | | |  | |  | |  | |  | |  | |  | |
| --- | --- | --- | --- | --- | --- | --- | --- | --- | --- | --- | --- | --- | --- | --- | --- | --- | --- | --- | --- | --- | --- |
| Gene | **High 6h vs**  **Wind 6h** | | **High 24h vs**  **High 6h** | | | **High 30h vs**  **High 24h** | | | **Low 6h vs**  **Wind 6h** | | | | **Low 24h vs**  **Low 6h** | | | | **Low 30h vs**  **Low 24h** | | | |  |
|  | log2FC | P(adj) | log2FC | | P(adj) | log2FC | | P(adj) | log2FC | | P(adj) | | log2FC | | P(adj) | | log2FC | | P(adj) | |  |
| *Dhcr24* | **2.96** | 0.009 | **-2.87** | | 0.000 | 1.56 | | 0.393 | -0.06 | | 0.999 | | 2.07 | | 0.398 | | **-2.62** | | 0.041 | |  |
| *FDFT1* | **2.53** | 0.038 | **-2.74** | | 0.000 | 1.86 | | 0.146 | -1.87 | | 0.760 | | **2.95** | | 0.003 | | -1.56 | | 0.221 | |  |
| *Tspo* | **5.48** | 0.000 | **-2.98** | | 0.000 | 1.16 | | 0.609 | -0.33 | | 1.000 | | 3.78 | | 0.054 | | **-4.67** | | 0.041 | |  |
| *Scp2d1* | 5.36 | 0.475 | -5.45 | | 0.280 | – | | – | – | | – | | – | | – | | – | | – | |  |
| *Cyp17a1* | **5.65** | 0.002 | **-3.50** | | 0.024 | 0.34 | | 1.000 | -0.56 | | 1.000 | | 0.80 | | 1.000 | | -0.73 | | 1.000 | |  |
| *Hsd17b11* | **5.01** | 0.000 | **-3.68** | | 0.000 | 1.59 | | 0.468 | -1.57 | | 1.000 | | **5.28** | | 0.007 | | **-4.83** | | 0.030 | |  |
| *Hsd17b12* | **4.14** | 0.000 | **-2.32** | | 0.000 | 0.92 | | 0.678 | -0.47 | | 0.998 | | 3.38 | | 0.118 | | -3.72 | | 0.111 | |  |
| *Cyb5r1* | 1.54 | 0.435 | **-2.00** | | 0.000 | **1.54** | | 0.024 | -3.58 | | 0.356 | | **5.45** | | 0.000 | | **-2.85** | | 0.043 | |  |
| *Sc5d* | **3.41** | 0.000 | **-2.81** | | 0.000 | 1.02 | | 0.796 | -3.01 | | 1.000 | | **5.20** | | 0.003 | | **-4.08** | | 0.029 | |  |
| *Scp2* | **4.11** | 0.000 | **-2.33** | | 0.000 | 0.81 | | 0.681 | -0.40 | | 0.998 | | **2.90** | | 0.028 | | **-3.00** | | 0.012 | |  |
| *Cyb5r3* | **4.60** | 0.000 | **-2.17** | | 0.001 | 0.66 | | 0.904 | -1.86 | | 0.942 | | **5.33** | | 0.029 | | -3.98 | | 0.061 | |  |
| *Tecr* | **4.21** | 0.000 | **-2.67** | | 0.000 | 1.02 | | 0.658 | -0.09 | | 0.998 | | 2.91 | | 0.190 | | **-4.19** | | 0.018 | |  |
| *Hmgcs1* | **3.57** | 0.000 | **-3.29** | | 0.000 | 2.02 | | 0.174 | -1.22 | | 0.977 | | 3.14 | | 0.051 | | **-2.59** | | 0.043 | |  |
| *Msmo1* | **2.82** | 0.025 | **-3.10** | | 0.000 | 1.16 | | 0.451 | -1.89 | | 0.846 | | **3.22** | | 0.008 | | -2.13 | | 0.060 | |  |
| **male gonad development (GO:0008584)** | | | |  | |  |  | | |  | |  | |  | |  | |  | |  | |

| Gene | **High 6h vs**  **Wind 6h** | | **High 24h vs**  **High 6h** | | **High 30h vs**  **High 24h** | | **Low 6h vs**  **Wind 6h** | | **Low 24h vs**  **Low 6h** | | **Low 30h vs**  **Low 24h** | |
| --- | --- | --- | --- | --- | --- | --- | --- | --- | --- | --- | --- | --- |
|  | log2FC | P(adj) | log2FC | P(adj) | log2FC | P(adj) | log2FC | P(adj) | log2FC | P(adj) | log2FC | P(adj) |
| *Insl6* | **5.53** | 0.018 | **-4.53** | 0.013 | 1.43 | 1.000 | – | – | 3.56 | 1.000 | -3.49 | 1.000 |
| *BCL2L1* | **3.01** | 0.001 | **-2.10** | 0.000 | **1.73** | 0.020 | -2.38 | 0.658 | **4.54** | 0.000 | **-2.92** | 0.009 |
| *Cited2* | **3.26** | 0.006 | -1.29 | 0.009 | **1.99** | 0.021 | -1.65 | 0.910 | **3.81** | 0.000 | **-2.85** | 0.004 |
| *Ybx3* | **5.16** | 0.000 | **-3.54** | 0.000 | 1.45 | 0.099 | 0.66 | 0.995 | 1.92 | 0.289 | -1.85 | 0.237 |
| *Kdr* | **5.40** | 0.000 | **-3.49** | 0.000 | 1.43 | 0.854 | -0.58 | 1.000 | 3.25 | 0.395 | -3.80 | 1.000 |
| *Eif2s2* | **2.66** | 0.032 | **-2.13** | 0.000 | 1.25 | 0.221 | -1.38 | 0.925 | **3.19** | 0.004 | -1.95 | 0.174 |
| *Tbc1d20* | **3.56** | 0.000 | **-3.76** | 0.000 | 1.02 | 0.390 | -1.69 | 0.799 | **4.33** | 0.000 | **-3.10** | 0.035 |
| *Wdr48* | **4.82** | 0.000 | **-3.86** | 0.000 | 1.34 | 0.439 | -2.22 | 1.000 | **4.24** | 0.007 | **-3.76** | 0.017 |
| *PRPS1* | 3.91 | 0.635 | -3.99 | 1.000 | – | – | – | – | – | – | – | – |
| *CSDE1* | **2.38** | 0.007 | **-2.33** | 0.000 | 1.48 | 0.092 | -1.43 | 0.836 | **2.55** | 0.019 | -1.79 | 0.175 |
| *Klhl10* | **21.56** | 0.000 | **-22.41** | 0.000 | 0.34 | 1.000 | – | – | – | – | – | – |
| *ANKRD7* | 4.93 | 0.543 | -5.02 | 0.337 | – | – | – | – | – | – | – | – |
| *Spink2* | 5.95 | 0.380 | -6.03 | 0.211 | 0.34 | 1.000 | – | – | – | – | – | – |
| *Nupr1* | 1.77 | 0.297 | **-2.14** | 0.000 | 1.52 | 0.185 | -2.27 | 0.885 | 3.61 | 0.062 | -2.50 | 0.371 |
| *Rbp4* | **7.05** | 0.000 | -1.01 | 0.542 | 0.18 | 0.995 | 0.52 |  | 2.01 | 1.000 | -2.65 | 1.000 |
| *Insl3* | **7.19** | 0.002 | **-6.92** | 0.000 | 1.46 | 1.000 | – | – | 0.80 | 1.000 | -0.73 | 1.000 |
| *Prdx4* | 2.31 | 0.090 | **-2.16** | 0.000 | 1.06 | 0.619 | -2.11 | 0.759 | **3.90** | 0.000 | -1.67 | 0.358 |
| *PRDX4* | 5.50 | 0.453 | -5.58 | 0.264 | 0.00 | 1.000 | – | – | – | – | – | – |
| *Fdps* | 2.53 | 0.079 | **-2.74** | 0.000 | 1.13 | 0.554 | -2.09 | 0.924 | **3.75** | 0.045 | -2.66 | 0.302 |
| *Bax* | **2.68** | 0.006 | **-1.95** | 0.000 | 0.98 | 0.328 | -2.03 | 0.699 | **4.44** | 0.000 | **-3.53** | 0.009 |
| *Hmgb2* | **2.59** | 0.073 | **-3.72** | 0.000 | 1.74 | 0.109 | -2.18 | 0.720 | **2.61** | 0.044 | -1.19 | 0.566 |
| *SFRP2* | 2.46 | 0.158 | 1.34 | 0.060 | -2.26 | 0.125 | -3.09 | 1.000 | **5.20** | 0.000 | -1.93 | 0.298 |
| *Six4* | -0.30 | 0.220 | 0.47 | 0.006 | -0.01 | 0.996 | 0.27 | 0.720 | -0.14 | 0.733 | 0.36 | 0.084 |
| *CITED2* | **3.77** | 0.000 | **-1.73** | 0.000 | **2.01** | 0.020 | -1.09 | 1.000 | **3.42** | 0.001 | **-2.44** | 0.030 |
| *NASP* | **1.96** | 0.419 | **-3.06** | 0.000 | 0.09 | 0.991 | -2.31 | 0.708 | **2.58** | 0.031 | -1.06 | 0.738 |

| **response to hormone (GO:0009725)** |  |  |  |  |  |  |  |  |  |
| --- | --- | --- | --- | --- | --- | --- | --- | --- | --- |

| Gene | **High 6h vs**  **Wind 6h** | | **High 24h vs**  **High 6h** | | **High 30h vs**  **High 24h** | | **Low 6h vs**  **Wind 6h** | | **Low 24h vs**  **Low 6h** | | **Low 30h vs**  **Low 24h** | |
| --- | --- | --- | --- | --- | --- | --- | --- | --- | --- | --- | --- | --- |
|  | log2FC | P(adj) | log2FC | P(adj) | log2FC | P(adj) | log2FC | P(adj) | log2FC | P(adj) | log2FC | P(adj) |
| *Timp2* | 2.07 | 0.278 | **-2.48** | 0.000 | 0.95 | 0.785 | -2.32 | 0.854 | **4.56** | 0.006 | **-3.21** | 0.022 |
| *Mmp14* | 1.49 | 0.535 | -0.71 | 0.400 | 1.25 | 0.339 | -2.23 | 0.769 | **4.30** | 0.001 | -2.16 | 0.162 |
| *Lox* | 2.26 | 0.235 | **-2.78** | 0.000 | 1.64 | 0.572 | -4.15 | 0.648 | **5.58** | 0.005 | -2.62 | 0.413 |
| *Mb* | **6.49** | 0.000 | -0.83 | 0.786 | 1.42 | 0.957 | 2.93 | 1.000 | -2.69 | 1.000 | – | – |
| *GPX1* | 0.50 | 1.000 | -0.58 | 1.000 | – | – | – | – | 1.16 | 1.000 | -1.09 | 1.000 |
| *Cox5b* | **5.33** | 0.000 | **-2.68** | 0.000 | 1.05 | 0.661 | 0.03 | 1.000 | 3.32 | 0.114 | -3.56 | 0.093 |
| *Mtnd3* | **6.60** | 0.000 | **-3.39** | 0.000 | 1.19 | 0.722 | 1.72 | 0.914 | 1.24 | 0.702 | -2.19 | 0.419 |
| *HCLS1* | **4.61** | 0.000 | **-2.03** | 0.000 | 0.93 | 0.369 | -0.03 | 0.999 | -0.79 | 0.761 | -0.30 | 0.948 |
| *SORD* | **3.49** | 0.008 | **-3.70** | 0.000 | 1.27 | 0.316 | -1.55 | 0.902 | **3.42** | 0.003 | **-2.94** | 0.022 |
| *NCOA4* | **4.94** | 0.000 | **-3.18** | 0.000 | 1.14 | 0.621 | -1.05 | 0.993 | 4.26 | 0.134 | **-4.55** | 0.031 |
| *Timp1* | 2.66 | 0.075 | **-2.71** | 0.001 | 1.39 | 0.585 | -5.57 | 0.142 | **7.30** | 0.000 | -2.99 | 0.319 |
| *Me1* | **3.96** | 0.000 | **-2.66** | 0.000 | 0.93 | 0.697 | -0.39 | 0.998 | 2.33 | 0.176 | -2.21 | 0.054 |
| *Aqp1* | **6.78** | 0.000 | **-2.25** | 0.004 | -0.02 | 0.998 | 4.04 | 1.000 | -3.45 | 1.000 | 2.48 | 1.000 |
| *Por* | **3.97** | 0.000 | **-2.74** | 0.000 | 1.29 | 0.074 | -0.83 | 0.956 | 1.54 | 0.159 | -1.16 | 0.353 |

**Table S3**: Food type, volumes, and nutritional information of the food fed to boreal chorus frog (*Pseudacris maculata*) tadpoles in experiment to determine the effect of broadcast of conspecific calls on the reproductive output and offspring quality.

| Every 2 days   - 90 g of Sera Micron Nature - 4 mL of Seachem Reef Phytoplankton - 20 mL of rotifers (supplied by uOttawa ACVS facility) - 6 g of ground Nasco Adult Frog Brittle   Every 7 days   - 0.6 g of boiled spinach | |
| --- | --- |
| Sera Micron Nature  (SKU: 00720) | Ingredients:  spirulina (51%), krill (18%), brine shrimps, fish meal, wheat flour, stinging nettle, herbs, alfalfa, brewers yeast, parsley, sea algae, paprika, Ca-caseinate, gammarus, cod-liver oil (containing 34% omega fatty acids), spinach, carrots, mannan oligosaccharides, Haematococcus algae, green-lipped mussel, garlic.  Guaranteed Analysis:  Protein (min): 55.6%  Crude Fat (min): 6.2%  Crude Fiber (max): 11.4%  Moisture (max): 7.0%  Ash (max): 11.3%  Additives:  Vitamins and provitamins: Vit. A 2,800 IU/lb., Vit. D3 135 IU/lb., Vit. E (D, L-α-tocopheryl acetate) 9 IU/lb., Vit. B1 2.7 mg/lb., Vit. B2 6.8 mg/lb., Stabilized Vit. C (L-ascorbyl monophosphate) 41 mg/lb. |
| Seachem Reef Phytoplankton  (SKU: 20343) | Ingredients:  Water, Sodium chloride, Thalassiosira weissflogii, Acetic Acid, Isochrysis sp, Ascorbic Acid, Citric Acid, Nannochloropsis, Astaxanthin  Guaranteed Analysis:  Protein (min): 0.40%  Crude Fat (min): 0.15%  Crude Fiber (max): 0.5%  Moisture (max): 96.8%  Ash (max): 3.0% |
| Nasco Adult Frog Brittle  (SKU: SA05961[LM]) | Ingredients:  Fish meal, porcine meat and bone meal, dehulled soybean meal, ground corn, wheat flour, brewers dried yeast, dried egg  product, glyceryl monostearate, corn distillers dried grains with solubles, whey, wheat germ, salt, choline chloride, pyridoxine  hydrochloride, l-ascorbyl-2-polyphosphate (stabilized vitamin C), dl-alpha tocopheryl acetate (form of vitamin E), biotin,  cholecalciferol (form of vitamin D3), vitamin A acetate, calcium carbonate, calcium pantothenate, menadione sodium bisulfite  complex (source of vitamin K), ethoxyquin (a preservative), thiamine mononitrate, folic acid, riboflavin supplement, nicotinic acid, vitamin B12 supplement, manganous oxide, zinc oxide, ferrous carbonate, copper sulfate, zinc sulfate, calcium iodate, cobalt carbonate, sodium selenite.  Guaranteed Analysis:  Protein (min): 44.0%  Crude Fat (min): 6.0%  Crude Fiber (max): 5.0%  Moisture (max): 12.0%  Ash (max): 15% |
